# A Nuclear Calcium-Sensing Pathway Is Critical for Gene Regulation and Salt Stress Tolerance in *Arabidopsis*

**DOI:** 10.1371/journal.pgen.1003755

**Published:** 2013-08-29

**Authors:** Qingmei Guan, Jianmin Wu, Xiule Yue, Yanyan Zhang, Jianhua Zhu

**Affiliations:** Department of Plant Science and Landscape Architecture, University of Maryland, College Park, Maryland, United States of America; Institute of Natural Resources and Agrobiology, CSIC, Spain

## Abstract

Salt stress is an important environmental factor that significantly limits crop productivity worldwide. Studies on responses of plants to salt stress in recent years have identified novel signaling pathways and have been at the forefront of plant stress biology and plant biology in general. Thus far, research on salt stress in plants has been focused on cytoplasmic signaling pathways. In this study, we discovered a nuclear calcium-sensing and signaling pathway that is critical for salt stress tolerance in the reference plant *Arabidopsis*. Through a forward genetic screen, we found a nuclear-localized calcium-binding protein, RSA1 (SHORT ROOT IN SALT MEDIUM 1), which is required for salt tolerance, and identified its interacting partner, RITF1, a bHLH transcription factor. We show that RSA1 and RITF1 regulate the transcription of several genes involved in the detoxification of reactive oxygen species generated by salt stress and that they also regulate the *SOS1* gene that encodes a plasma membrane Na^+^/H^+^ antiporter essential for salt tolerance. Together, our results suggest the existence of a novel nuclear calcium-sensing and -signaling pathway that is important for gene regulation and salt stress tolerance.

## Author Summary

Salt stress greatly constrains the productivity of crops worldwide. With the increasing demand for food and fiber from the growing world population, sustainability of crop production is of fundamental importance. Studies with plants in the past decade have identified novel signaling pathways, and so far, research on salt stress in plants has focused on cytoplasmic signaling pathways. In the current study, we revealed a nuclear calcium-sensing and -signaling pathway that is essential for salt tolerance in *Arabidopsis*. We identified a nuclear-localized calcium-binding protein, RSA1, in a forward genetic screen for salt tolerance determinants. We found an RSA1 interacting partner, RITF1, a bHLH transcription factor. RSA1 and RITF1 regulate the transcription of several genes involved in the scavenging of reactive oxygen species that are caused by salt stress, and they also regulate the *SOS1* gene that encodes a plasma membrane-localized Na^+^/H^+^ antiporter crucial for Na^+^ homeostasis and salt tolerance. Our research suggests a novel nuclear calcium-sensing and -signaling pathway is important for gene regulation and salt stress tolerance.

## Introduction

Salt stress severely limits the quality and productivity of important agricultural crops worldwide. Therefore, a thorough understanding of the molecular basis of salt stress signal transduction pathways and salt tolerance mechanisms is of fundamental importance for the understanding of plant biology and for the generation of salt-tolerant crops through rational breeding and genetic engineering strategies.

High levels of soluble salts including chlorides of sodium, calcium, and magnesium often cause soil sodicity, alkalinity, and other soil problems. Excessive salts in soil are harmful to plants because of toxicity of Na^+^ and other ions [1]. To deal with salt stress, plants have evolved mechanisms in order to coordinate the activities of various ion transporters and to thereby maintain ion homeostasis in the cell cytoplasm. Salt Overly Sensitive (SOS) pathway proteins play a key role in Na^+^ homeostasis in plants [2]. The calcium sensor SOS3 senses salt stress-induced increases of cytosolic free calcium levels and interacts with and activates a serine/threonine protein kinase SOS2 [2]. The SOS3-SOS2 protein kinase complex then phosphorylates and thereby activates the plasma membrane-localized Na^+^/H^+^ antiporter SOS1 [2]. Overexpression of *SOS1* leads to improved salt tolerance in transgenic *Arabidopsis* [2], [3]. SOS2 also positively controls the activities of tonoplast Na^+^/H^+^ antiporters, which sequester Na^+^ ions in the vacuole, and the activities of a vacuolar H^+^/Ca^2+^ exchanger CAX1 [4], [5]. In addition to its critical role in Na^+^ homeostasis, SOS1 is important for oxidative stress responses under salt stress [6].

Salt stress and many other abiotic and biotic stresses cause plants to produce reactive oxygen species (ROS) including superoxide (O_2_^.−^) and hydroxyl (OH^.^) free radicals, hydrogen peroxide (H_2_O_2_), and free singlet oxygen [7]–[9]. In many cases, the over-accumulated ROS have detrimental effects on cellular processes [10]. Below the threshold level that is not harmful to plant cells, however, H_2_O_2_ can act as a messenger molecule to initiate signal transduction cascades involving mitogen-activated protein kinase (MAPK) under various environmental cues [11], [12]. Plants have developed enzymatic (e.g., superoxide dismutase, peroxidase, and catalase) and non-enzymatic (e.g., antioxidants and some secondary metabolites) strategies to detoxify excessive ROS [7]–[9].

Here, we report the isolation and characterization of a nuclear-localized calcium-binding protein, RSA1, which was identified in a forward genetic screen for critical genes required for salt tolerance in plants. The *rsa1-1* mutant plants are hypersensitive to NaCl but not to LiCl, CsCl, or general osmotic stress. The *rsa1-1* plants over-accumulate ROS and are hypersensitive to exogenous H_2_O_2_ or ROS-producing agents such as methyl viologen. Protein-protein interaction studies revealed that RSA1 interacts with a bHLH transcription factor, RITF1, which in turn regulates many RSA1 downstream target genes, including *SOS1*, that are important for ROS detoxification and/or Na^+^ homeostasis. Together, our results demonstrate that RSA1 is a nucleus-localized calcium sensor that has a crucial role in gene regulation under salt stress and in salt stress tolerance.

## Results

### Identification of the *rsa1-1* mutant

**Figure 1:**
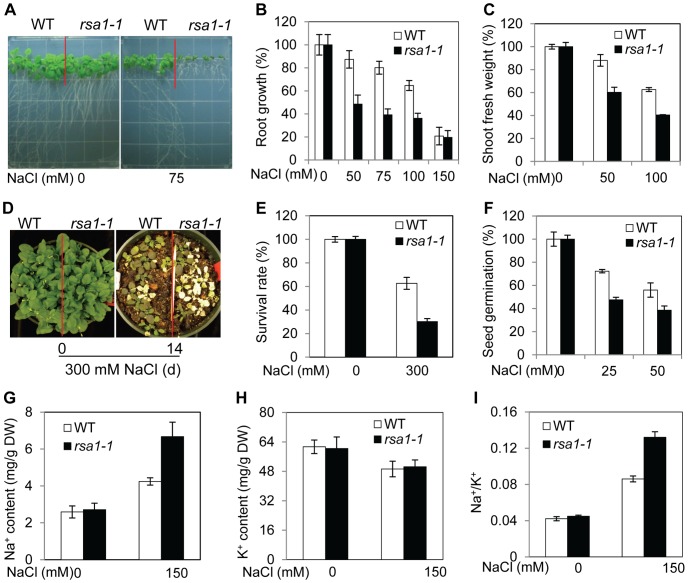
*rsa1-1* plants are hypersensitive to NaCl, and RSA1 is involved in Na^+^ homeostasis under salt stress. (A)–(C) Five-d-old wild-type and *rsa1-1* seedlings grown on MS medium were transferred to MS medium supplemented with different levels of NaCl and allowed to grow for an additional 8 d. Root elongation or shoot fresh weight was measured and is shown as a percentage relative to growth on normal MS medium. (D) Two-week-old wild-type and *rsa1-1* plants grown in soil were irrigated with 300 mM NaCl for 0 or 14 d. (E) Survival rate of wild-type and *rsa1-1* plants as shown in (D). (F) Seed germination of wild type and *rsa1-1* in response to various NaCl levels. There were 80–150 seeds per genotype per biological replicate. Seeds in which the radical had emerged through the seed coat were considered germinated. (G) Na^+^ content in soil-grown wild-type and *rsa1-1* plants. DW, dry weight. (H) K^+^ content in soil-grown wild-type and *rsa1-1* plants. (I) Ratio of Na^+^ to K^+^ accumulation in soil-grown wild-type and *rsa1-1* plants. WT, wild type. Error bars indicate the standard deviation (n = 30–40). The experiments in Figure 1 were repeated at least five times with similar results, and data from one representative experiment are presented.

To isolate genes that play essential roles in salt stress tolerance, we used a modified root-bending assay [13], [14] and screened an ethyl methanesulfonate (EMS)-mutagenized *Arabidopsis* M_2_ population for mutants with hypersensitivity to 100 mM NaCl. These mutants were designated as *short root in salt medium* (*rsa*). One of these mutants, *rsa1-1*, was chosen for detailed characterization. The shoot development of *rsa1-1* is normal under control conditions, but the primary root of *rsa1-1* is slightly shorter than that of the wild type (Figure 1A). Both roots and shoots of *rsa1-1* seedlings display a hypersensitive phenotype in response to supplemental NaCl in the growth medium (Figure 1A–1C). The roots of *rsa1-1* seedlings have more root hairs than the wild type under control and salt-treated conditions (Figure S1A). The soil-grown *rsa1-1* plants are more sensitive than the wild type when treated with 300 mM NaCl (Figure 1D and 1E), and germination of *rsa1-1* seeds is more inhibited by NaCl than is germination of wild-type seeds (Figure 1F), suggesting that salt hypersensitivity of *rsa1-1* plants does not depend on developmental stage. We then determined whether the salt hypersensitive phenotype of *rsa1-1* is specific to Na^+^. We found that *rsa1-1* mutant plants are not more sensitive to LiCl or CsCl than the wild type (Figure S1B and S1C), even though Li and Cs are in the same column of the periodic table of the elements as Na and are generally considered more toxic. The response of *rsa1-1* plants to mannitol, which induces general osmotic stress, was similar to that of wild-type plants (Figure S1D). Thus, *rsa1-1* is only hypersensitive to NaCl.

When *rsa1-1* plants were backcrossed with wild-type plants, all F_1_ plants displayed a wild-type phenotype, and F_2_ progeny from the self-pollinated F_1_ plants segregated at approximately 3∶1 (wild type vs. mutant; Table S1). These results suggest that *rsa1-1* is a recessive mutation in a single nuclear gene.

### The *rsa1-1* mutation affects Na^+^ homeostasis

The altered responses to salt stress in *rsa1-1* suggest that ion homeostasis in *rsa1-1* may be disturbed. As shown in Figure 1G, soil-grown *rsa1-1* plants accumulate more Na^+^ than the wild type when treated with 150 mM NaCl. Relative to wild-type plants, the K^+^ level is not altered in *rsa1-1* with or without salt stress (Figure 1H). The ratio of Na^+^ to K^+^ is much higher in *rsa1-1* than in the wild type under salt stress (Figure 1I). These results suggest that RSA1 is required for Na^+^ homeostasis under salt stress. These results also suggest that RSA1 may directly or indirectly affect the known SOS pathway for Na^+^ homeostasis under salt stress.

### The *rsa1-1* mutation causes over-accumulation of ROS

**Figure 2:**
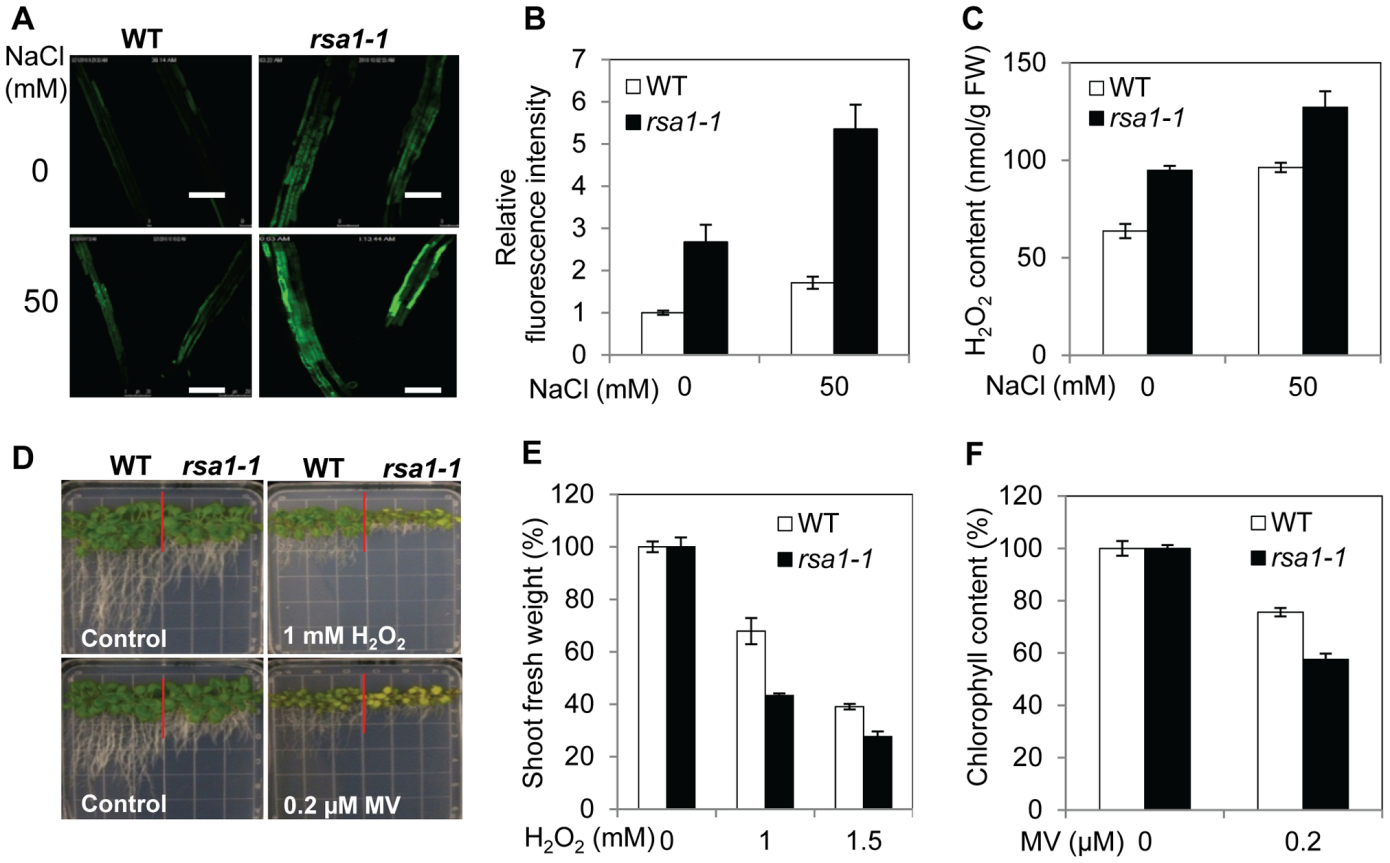
*rsa1-1* mutant plants accumulate more ROS and are hypersensitive to oxidative stress. (A) Total ROS accumulation in roots of 5-d-old wild-type and *rsa1-1* seedlings subjected to 0 or 50 mM NaCl for 12 h. Bars = 200 µm. (B) Fluorescence intensity in plants shown in (A). (C) H_2_O_2_ content of wild-type and *rsa1-1* plants subjected to 0 or 50 mM NaCl for 12 h. (D) Growth responses of wild-type and *rsa1-1* seedlings to oxidative stress-inducing reagents H_2_O_2_ and methyl viologen (MV). Seeds of the wild type and *rsa1-1* were sown directly on MS medium containing 0 or 1 mM H_2_O_2_ (upper panel) and 0 or 0.2 µM MV (lower panel) and allowed to grow for an additional 14 d. (E) and (F) Shoot fresh weight and chlorophyll content in wild-type and *rsa1-1* plants under treatment conditions shown in (D). Error bars indicate the standard deviation (n = 24). The experiments in Figure 2 were repeated at least four times with similar results, and data from one representative experiment are presented.

Abiotic stresses including salt stress can cause production of ROS [7], [15]. We determined the effect of the *rsa1-1* mutation on ROS levels and on the response to oxidative stress. The fluorescent dye 5-(and 6)-chloromethyl-2′7′-dichlorodihydrofluorescein diacetate acetyl ester (CM-H_2_DCFDA) was used to visualize and quantify total ROS in the root tissues. The *rsa1-1* plants accumulated slightly more ROS than the wild type under control conditions but accumulated substantially more ROS than the wild type when treated with 50 mM NaCl (Figure 2A and 2B). Similar results were obtained for the levels of hydrogen peroxide (H_2_O_2_), which was quantified with the Amplex red reagent, 10-acetyl-3,7-dihydrophenoxazine (Figure 2C). These results suggest that RSA1 is an important regulator of ROS accumulation in plants under salt stress. Furthermore, we found that *rsa1-1* plants are hypersensitive to exogenous application of H_2_O_2_ or methyl viologen (MV) (Figure 2D–2F). MV can lead to an increase in the generation of toxic superoxide free radicals in chloroplasts [16].

### *RSA1* encodes a nuclear protein containing an EF-hand motif

We identified the *RSA1* locus through a map-based cloning strategy. The *rsa1-1* mutation is caused by a single nucleotide substitution in *At2g03150*, and this mutation results in the change of proline at position of 685 to leucine in the deduced RSA1 polypeptide (Figure S2A). *RSA1* encodes a putative calcium-binding protein with one EF-hand motif at its C-terminal region (Figure S2A). We confirmed the identity of *RSA1* by a gene complementation analysis. The wild-type *RSA1* driven by its native promoter is able to fully complement the *rsa1-1* mutant phenotype (Figure S2B and S2C). Two T-DNA alleles of *RSA1* (*rsa1-2* and *rsa1-3*) were obtained, and expression of *RSA1* is not detectable in either *rsa1-2* or *rsa1-3* plants, suggesting that they are null alleles of *RSA1* (Figure S2D). Like *rsa1-1* plants, *rsa1-2* and *rsa1-3* plants are hypersensitive to NaCl stress (Figure S2B and S2E).

**Figure 3:**
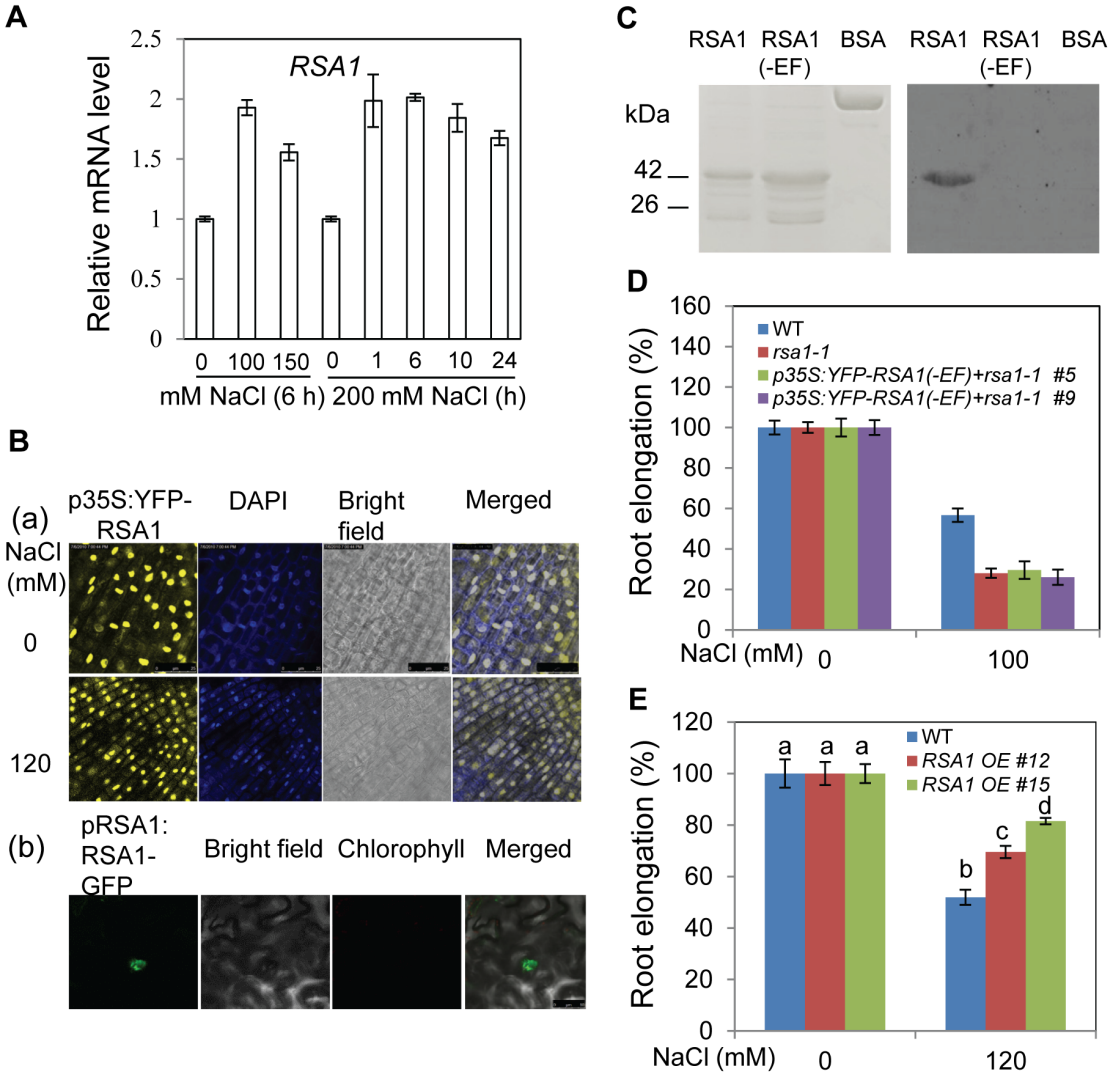
RSA1 is localized in the nucleus and has calcium-binding activity. (A) *RSA1* expression under salt stress. Fourteen-d-old wild-type seedlings grown on MS medium were transferred to filter paper saturated with 0, 100, 150, and 200 mM NaCl for the indicated time. (B) Localization of p35S:YFP-RSA1 in *Arabidopsis* root tip cells with or without salt stress ([a], DAPI staining was used to indicate the nuclei) and pRSA1:RSA1-GFP in tobacco leaves (b). Bars = 25 µm in (a) and 50 µm in (b). (C) Calcium-binding activity of RSA1 and RSA1(-EF) proteins. 6× His-tagged proteins were purified, separated on SDS-PAGE, and stained with coomassie blue (left panel) or electroblotted onto a PVDF membrane, overlayed with ^45^Ca^2+^, and autoradiographed (right panel). BSA (bovine serum albumin), negative control for ^45^Ca^2+^ binding. (D) Salt tolerance of wild-type, *rsa1-1*, and *rsa1-1* plants expressing *p35S:YFP-RSA1(-EF)*. (E) Salt tolerance of *RSA1* overexpression plants in *rsa1-1* background (Figure S3F). Five-d-old wild-type and *rsa1-1* seedlings grown on MS medium were transferred to MS medium supplemented with different levels of NaCl and allowed to grow for an additional 8 d (for [D] and [E]). RSA1 (-EF), RSA1 without EF-hand motif. One-way ANOVA (Tukey-Kramer test) was performed, and statistically significant differences are indicated by different lowercase letters (p<0.01). Error bars represent the standard deviation (n = 4 in [A], 40 in [D] and [E]). The experiments in Figure 3 were performed at least three times with similar results, and data from one representative experiment are presented.

Strong expression of *RSA1* was detected in all examined tissues of wild-type plants including guard cells (Figure S3A and S3B). *RSA1* is slightly induced by salt stress (Figure 3A). Unlike most EF-hand proteins, RSA1 is predominantly localized in the nucleus of *Arabidopsis* root tip cells or tobacco leaf cells (Figure 3B). Upon salt stress, RSA1 continues to be localized in the nucleus (Figure 3B). The *p35S:YFP-RSA1* and *pRSA1:RSA1-GFP* genes used in our subcellular localization studies are able to restore the *rsa1-1* mutant phenotype to wild type (Figure S3C and S3D), suggesting that the *p35S:YFP-RSA1* and *pRSA1:RSA1-GFP* genes are functional *in planta*.

### RSA1 is a calcium-binding protein

Like other calcium-dependent protein kinases (CPKs) and calmodulin 2 (CAM2) in *Arabidopsis*, the EF-hand motif in RSA1 is highly conserved (Figure S3E) [17]. We produced a recombinant RSA1 protein with a 6× His tag at its N-terminus and purified it from *E. coli* to test its potential calcium-binding activity (Figure 3C). The recombinant RSA1 protein showed calcium-binding activity in the calcium overlay assays, and the calcium-binding activity was abolished when the 12-amino acid consensus sequence for calcium binding in the EF-hand motif was deleted (Figure 3C). We further showed that RSA1 protein carrying the 12-amino acid deletion in the EF-hand motif is unable to complement the *rsa1-1* mutant (Figure 3D), suggesting that the calcium-binding activity of RSA1 is required for its *in vivo* function. We were able to identify two independent lines of *Arabidopsis* transgenic plants overexpressing *RSA1* (*p35S:YFP-RSA1*) in the *rsa1-1* background (Figure S3F). These *RSA1* overexpression plants are substantially more tolerant to 120 mM NaCl than the wild type (Figure 3E). These results further confirm that RSA1 is a positive regulator of plant salt stress tolerance.

### RSA1 interacts with a basic helix-loop-helix (bHLH) transcription factor that is important for salt stress tolerance

**Figure 4:**
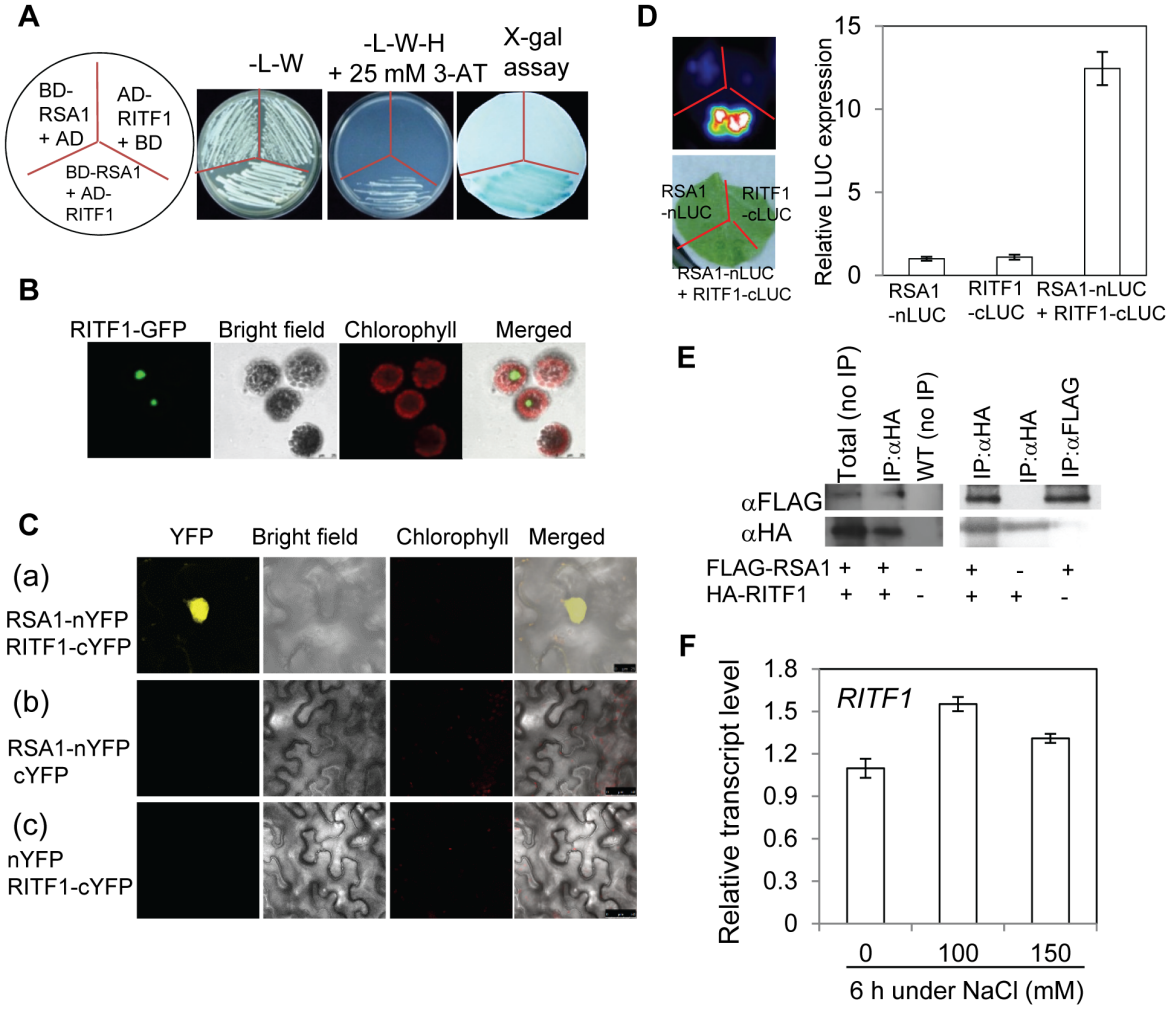
RSA1 interacts with RITF1. (A) RSA1 interacts with RITF1 as determined by yeast two-hybrid assays. Yeast strain AH109 co-transformed with RSA1-pDEST32 (bait) and RITF1-pDEST22 (prey) was subjected to x-gal assay. AH109 cells co-transformed with RSA1-pDEST32/pDEST22 (empty vector) or RITF1-pDEST22/pDEST32 (empty vector) were used as negative controls. Yeast cells grown on SD medium-L-W or SD medium-L-W-H+3-AT are shown. 3-AT, 3-amino-1,2,4-triazole. L, W, H, symbols for amino acids leucine, tryptophan, and histidine, respectively. SD, yeast minimal media. (B) Localization of RITF1-GFP in *Arabidopsis* protoplasts. Bar = 25 µm. (C) RSA1 interacts with RITF1 *in vivo* as determined by BiFC assays in tobacco leaf epidermal cells. Bars = 25 µm in (a), and 50 µm in (b) and (c). YFP images were detected at an approximate frequency of 4.04% (101 out of 2,501 tobacco leaf epidermal cells analyzed exhibited BiFC events). (D) RSA1 interacts with RITF1 *in vivo* as determined by Split-LUC assays. (E) RSA1 interacts with RITF1 *in vivo* as determined by Co-IP assays. (F) *RITF1* expression under salt stress. The qRT-PCR analysis was carried out with 14-d-old wild-type seedlings grown for 6 h on MS medium containing 0, 100, or 150 mM NaCl. Error bars represent the standard deviation (n = 20 in [D], 4 in [F]). The experiments in Figure 4 were performed at least three times with similar results, and data from one representative experiment are presented.

To determine how RSA1 functions in the salt stress tolerance pathway, we identified interaction partners of RSA1 via a yeast two-hybrid screen. Six RSA1 interacting proteins that are localized in the nucleus were identified in the yeast two-hybrid screen. One basic helix-loop-helix (bHLH) transcription factor, RITF1 (for RSA1 interacting transcription factor 1 encoded by *At3g06590*), appeared many times and showed the strongest interaction with RSA1 in our yeast two-hybrid screen (Figure 4A). We therefore focused on this transcription factor. We found that RITF1 is predominantly localized in the nucleus of *Arabidopsis* protoplasts (Figure 4B). Bimolecular fluorescence complementation (BiFC) analysis using *Arabidopsis* protoplasts and tobacco leaves, and split luciferase complementation (Split-LUC) analysis in tobacco leaves confirmed that RSA1 interacts with RITF1 *in vivo* (Figure 4C, 4D and Figure S4A). Co-immunoprecipitation (Co-IP) analysis in tobacco plants further confirmed the interaction between RSA1 and RITF1 *in vivo* (Figure 4E).

**Figure 5:**
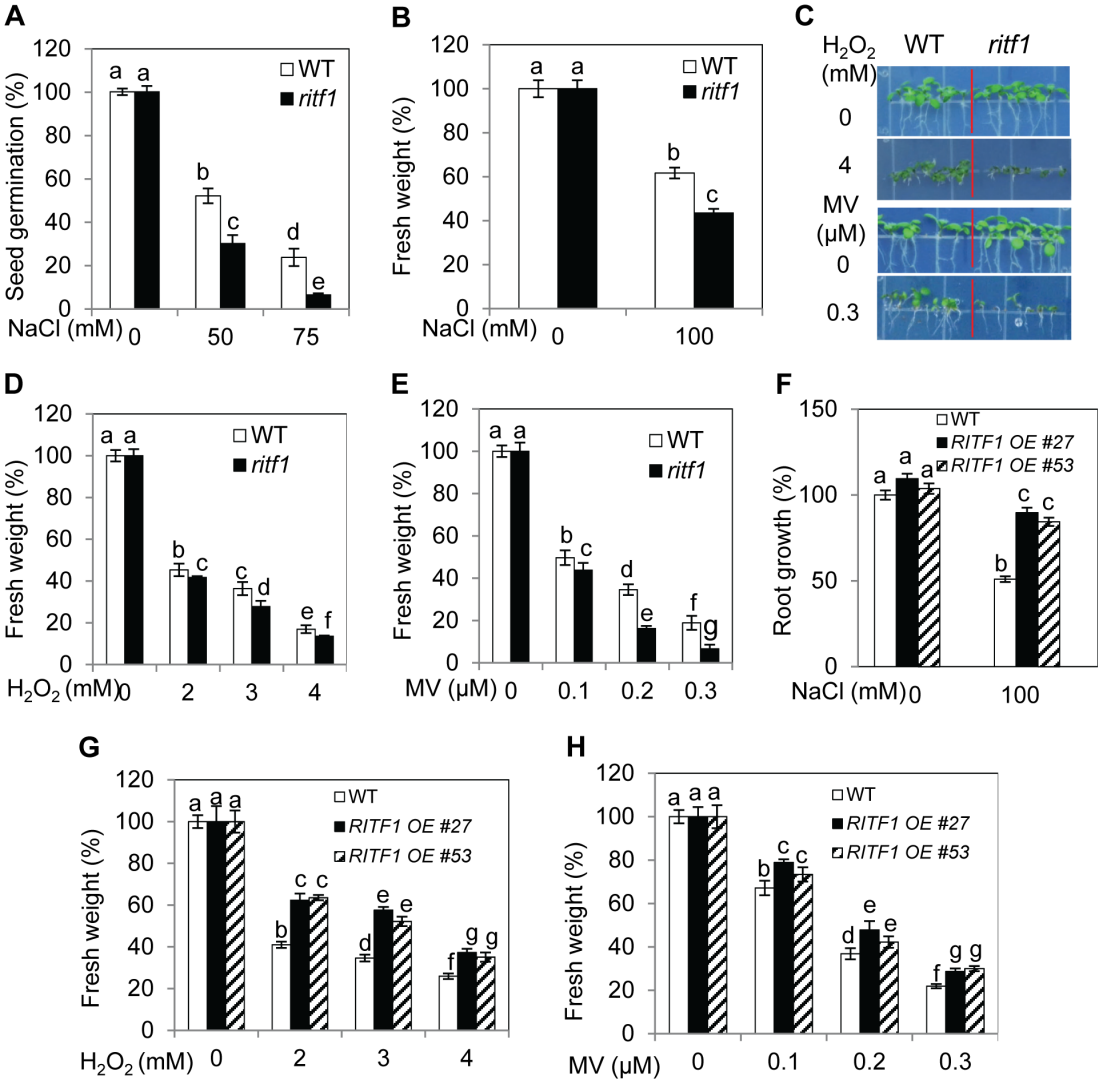
*ritf1* mutant plants are sensitive to salt and oxidative stresses, and overexpression of *RITF1* increases plant tolerance to salt and oxidative stresses. (A) Seed germination of wild type and *ritf1* in response to various NaCl levels. There were 80–150 seeds per genotype per biological replicate. (B) Fresh weight of wild-type and *ritf1* seedlings under salt stress. Five-d-old seedlings grown on MS medium were transferred to MS medium containing 0 or 100 mM NaCl and allowed to grow for an additional 7 d. (C) Growth responses of wild-type and *ritf1* seedlings to oxidative stress-inducing reagents H_2_O_2_ and methyl viologen (MV). (D) and (E) Fresh weight of seedlings grown on MS medium containing various levels of H_2_O_2_ (D) or MV (E) as shown in (C). (F) Salt tolerance of *RITF1* overexpression plants. Five-d-old seedlings grown on MS medium were transferred to MS medium containing 0 or 100 mM NaCl and allowed to grow for an additional 10 d. (G) and (H) Fresh weight of wild-type and *RITF1* overexpression plants grown on MS medium containing various levels of H_2_O_2_ (G) or MV (H). In (C)–(E), (G), and (H), seeds were sown directly on MS medium supplemented with various levels of H_2_O_2_ or MV and allowed to grow for an additional 10 d. Error bars represent the standard deviation (n = 8 in [A], 40 in [B], [D]–[H]). One-way ANOVA (Tukey-Kramer test) was performed, and statistically significant differences are indicated by different lowercase letters (p<0.05). The experiments in Figure 5 were repeated at least four times with similar results, and data from one representative experiment are presented.

qRT-PCR analysis indicated that *RITF1* is slightly inducible by salt stress (Figure 4F). We obtained the T-DNA knockdown plants of *RITF1* (*ritf1*) (Figure S4B). The *ritf1* plants are substantially more sensitive to salt stress than wild-type plants during seed germination and during seedling growth and development (Figure 5A and 5B). We further observed that *ritf1* plants are more sensitive to oxidative stress imposed by H_2_O_2_ or MV than the wild type (Figure 5C–5E). We overexpressed *RITF1* in *Arabidopsis* (Figure S4C), and all of the *RITF1* overexpression plants displayed increased tolerance to NaCl and the oxidative stress-inducing reagents H_2_O_2_ and MV (Figure 5F–5H). These results suggest that the RSA1 interacting transcription factor, RITF1, is required for plant tolerance to salt and oxidative stresses.

### RSA1 and RITF1 regulate the expression of genes important for oxidative and salt stress responses

**Figure 6:**
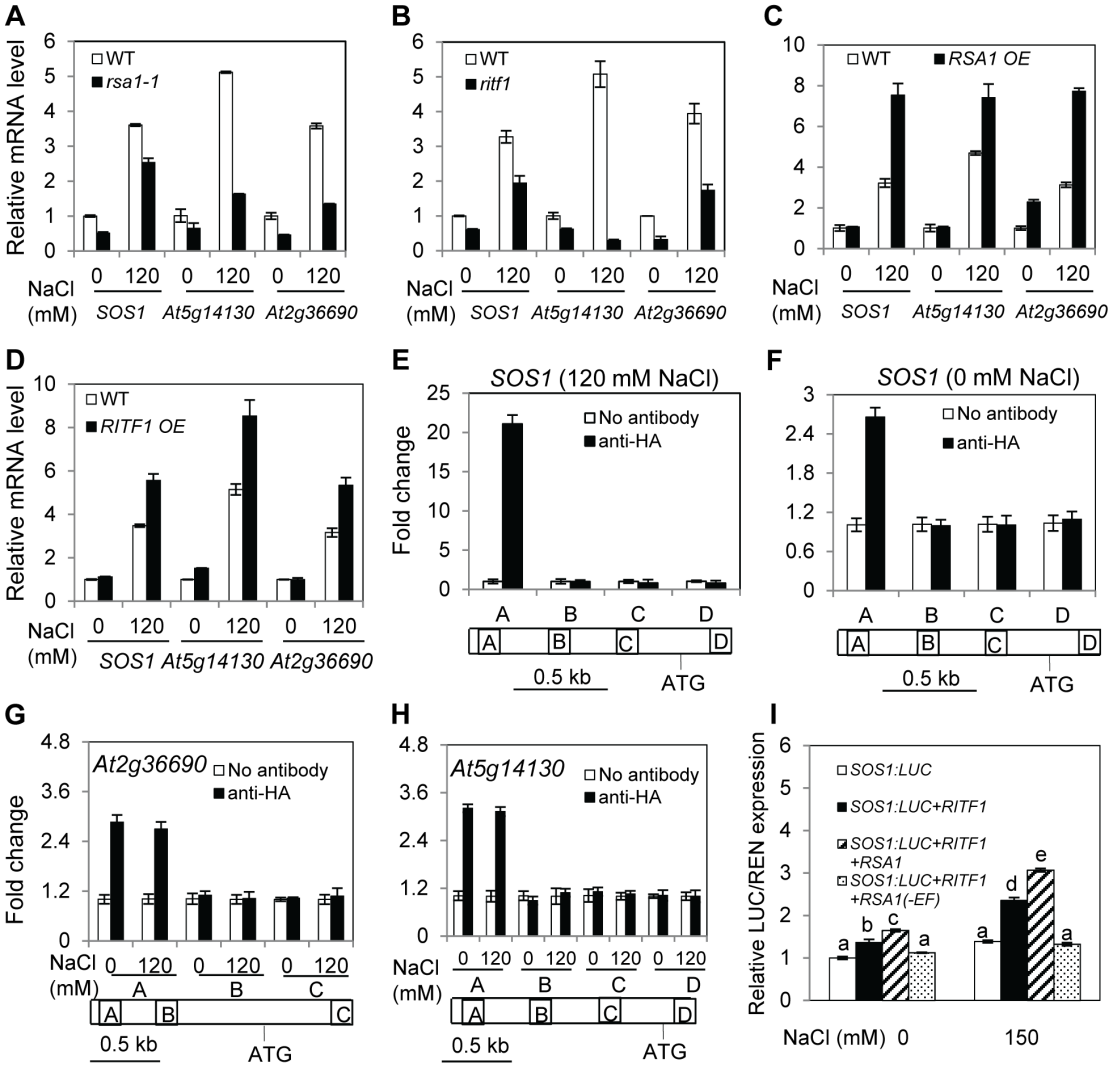
RSA1 and RITF1 regulate gene expression, and RITF1 binds directly to promoters of three RSA1 target genes. (A)–(D) Expression of *SOS1*, *At5g14130*, and *At2g36690* in wild-type, *rsa1-1*, *ritf1*, *RITF1* overexpression, or *RSA1* overexpression plants. *At5g14130* and *At2g36690* encode peroxidase superfamily protein, oxidoreductase (2-oxoglutarate (2OG) and Fe(II)-dependent oxygenase superfamily protein). Data in (C) and (D) are from multiple *RSA1 OE* or *RITF1 OE* plants (Figure S3F and S4C). Expression values from one representative transgenic line are presented. (E)–(H) ChIP-qPCR analysis of *SOS1*, *At2g36690*, and *At5g14130* genes. Regions of amplifications in (E)–(H) are specified in Table S6A. (I) Relative luciferase activity from the dual luciferase reporter assays in tobacco leaves. RSA1(-EF), RSA1 without EF-hand motif. Error bars represent the standard deviation (n = 4 in [A]–[H], 12 in [I]). One-way ANOVA (Tukey-Kramer test) was performed, and statistically significant differences are indicated by different lowercase letters (p<0.01). These experiments were repeated at least four times with similar results, and data from one representative experiment are presented.

Because RSA1 is localized in the nucleus, we determined whether the *rsa1-1* mutation affects gene expression. We performed a whole-genome microarray analysis with *Arabidopsis* Affymetrix ATH1 GeneChips. Compared to genes in the wild type, 41 genes displayed higher expression levels while 54 genes showed lower expression levels in *rsa1-1* by at least 2-fold (p<0.01) under control conditions (Table S2). The microarray analysis also revealed that 69 genes in *rsa1-1* displayed at least a 2-fold increase in transcripts levels while 76 genes in *rsa1-1* showed at least a 2-fold decrease in transcripts levels relative to the wild type (p<0.01) under salt stress conditions (Table S3). Compared to genes in the wild type, 13 genes displayed higher expression levels while 27 genes showed lower expression levels in *rsa1-1* by at least 2-fold (p<0.01) under both control and salt stress conditions (Table S4). The differentially expressed genes in *rsa1-1* encode proteins with diverse functions, and a large portion of these proteins have predicted functions in stress responses (Table S2, S3, and S4). In addition, as indicated by comparison with publicly available expression datasets, most of the differentially expressed genes in *rsa1-1* are not responsive to salt stress treatments in the wild type (Figure S5 and S6). We validated the microarray data with qRT-PCR analysis for four genes, which encode peroxidase, zinc finger protein 5, bHLH DNA-binding superfamily protein, and root hair specific 19 with putative peroxidase activity (Figure S7A–S7D). qRT-PCR analysis also revealed that the expression levels of *SOS1*, *At2g36690*, and *At5g14130* are dramatically reduced in *rsa1-1* under both control and salt stress conditions (Figure 6A), suggesting that RSA1 is a positive regulator of these three genes. *At2g36690* and *At5g14130* encode oxidoreductase and peroxidase, respectively. Therefore, RSA1 controls genes important for ROS detoxification and signal transduction. Furthermore, we observed that expression of *SOS1*, *At2g36690*, and *At5g14130* is substantially reduced in the *ritf1* mutant plants under both control and salt stress conditions (Figure 6B), indicating that similar to RSA1, RITF1 is a positive regulator of these three genes. Expression of *SOS1*, *At5g14130*, and *At2g36690* is substantially enhanced in transgenic plants overexpressing *RSA1* or *RITF1* under salt stress (Figure 6C and 6D), further confirming that RSA1 and RITF1 are positive regulators for salt-induced expression of *SOS1*, *At5g14130*, and *At2g36690*.

### RITF1 binds directly to *cis* elements in promoters of RSA1-regulated genes

bHLH transcription factors can potentially bind to a signature motif called E-box, which consists of a consensus hexanucleotide sequence of CANNTG [18], [19]. Database searches revealed that there are many such *cis* promoter elements in RSA1 downstream target genes as determined by the microarray analysis (Table S5). We then used a chromatin immunoprecipitation (ChIP) assay to determine whether RITF1 binds directly to these *cis* promoter elements *in vivo*. We produced transgenic *Arabidopsis* plants expressing the haemagglutinin (HA)-tagged RITF1 under the control of its native promoter (*RITF1:RITF1-HA*) (Figure S7E). RITF1 is enriched in the promoter regions of four genes (*SOS1*, *At2g36690*, *At5g14130*, and *At2g39040*) (Figure 6E–6H; Figure S7F). These results suggest that RSA1 controls expression of its downstream target genes through direct binding of RITF1 to promoters of RSA1 target genes.

We subsequently carried out a dual luciferase reporter assay to determine the effect of RSA1 and RITF1 on *SOS1* promoter activity *in vivo*. RSA1 and RITF1 can transiently activate the *SOS1* promoter that is transcriptionally fused with the firefly luciferase under both control and salt stress conditions (Figure 6I). When the EF-hand motif of RSA1 is deleted, the activation effect of RSA1 and RITF1 on the *SOS1* promoter is abolished (Figure 6I), suggesting that the calcium-binding activity of RSA1 is required for the transient activation of *SOS1* promoter activity. Furthermore, we observed that the *rsa1-2ritf1* double mutant plants do not display any additive effect of *rsa1-2* and *ritf1* mutations under salt stress or oxidative stress (Figure S8). Together, these results suggest that RSA1 and RITF1 function in a common pathway for gene expression and that both proteins are required for plant tolerance to salt and oxidative stresses.

## Discussion

By performing a forward genetic screen for genes crucial for salt stress tolerance, we have identified a salt hypersensitive mutant, *rsa1-1*. *RSA1* encodes a nuclear-localized calcium-binding protein that is important for gene regulation and salt stress tolerance.

**Figure 7:**
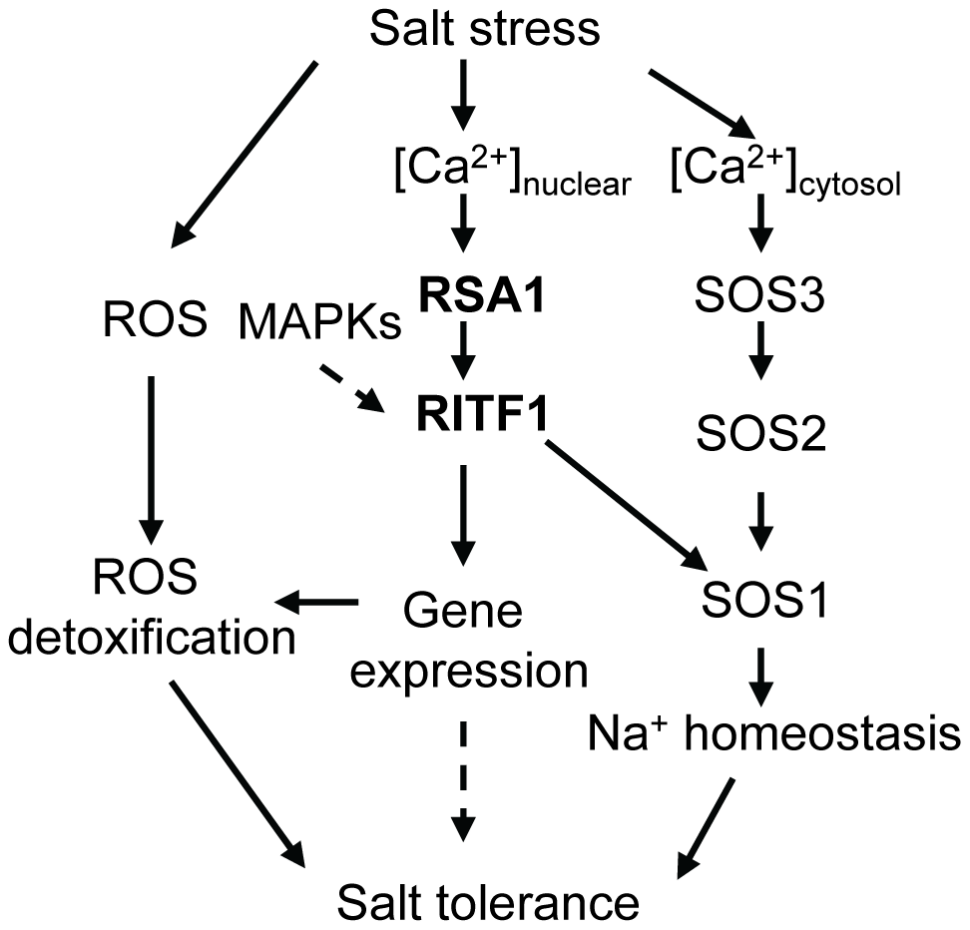
A working model for RSA1 and RITF1 function under salt stress. The calcium-binding protein RSA1 senses salt-induced changes in nuclear free calcium and interacts with a bHLH transcription factor, RITF1. RITF1 may be phosphorylated by nuclear-localized MAPKs. The RSA1-RITF1 complex controls expression of genes important for detoxification of salt-induced ROS and for Na^+^ homeostasis under salt stress. Some RITF1 target genes may play a role in salt tolerance with so far unknown mechanisms. The calcium-binding protein SOS3 senses salt-induced cytosolic calcium increases and interacts with SOS2, a protein kinase. The SOS3-SOS2 protein kinase complex then phosphorylates and thereby activates the plasma membrane-localized Na^+^/H^+^ antiporter SOS1.

Its location in the nucleus suggested that RSA1 might play a role in gene regulation, and this was supported by three other findings. First, the microarray analysis confirmed that RSA1 controls expression of a number of genes under both control and salt stress conditions (Table S2 and S3). A large portion of the differentially expressed genes in *rsa1-1* encode proteins with predicted roles in responses to abiotic or biotic stresses (Table S2 and S3). In addition, many genes in *rsa1-1* were differentially expressed under control conditions (as revealed in the microarray analysis, see Table S2), and these changes may contribute to the reduced growth of the primary root. This defect does not persist, however, because later stages of *rsa1-1* plants develop normally in potting soil. Second, yeast two-hybrid analysis indicated that RSA1 interacts with a bHLH transcription factor, RITF1. We confirmed this RSA1–RITF1 interaction *in vivo* by BiFC and Split-LUC assays and Co-IP analysis (Figure 4 and Figure S4A). Database searches indicate that RITF1 has three close homologs in *Arabidopsis* (bHLH transcription factors encoded by *At3g17100*, *At3g05800*, and *At1g09250*), and these homolog proteins do not interact with RSA1 in yeast two-hybrid assays (Figure S9A). T-DNA knockdown alleles of these three genes show normal morphology and the same responses to NaCl stress as the wild type (Figure S9B–S9E). These results suggest that RITF1 has a specialized role in salt stress responses. Interestingly, many of the RSA1 target genes revealed by the microarray analysis contain *cis*-promoter elements that RITF1 can potentially bind to (Table S5). Third, chromatin immunoprecipitation (ChIP) analysis indicated that RITF1 is able to bind to *cis*-promoter elements in four RSA1 target genes including *SOS1* (Figure 6E–6H; Figure S7F). This is the first time that two positive regulators (RSA1 and RITF1) for *SOS1* gene expression at the transcriptional level have been identified. Besides *SOS1*, the remaining three genes (*At5g14130*, *At2g36690*, and *At2g39040*) all encode enzymes for ROS detoxification. Thus, RSA1 is important for gene regulation through its interaction with partners such as the bHLH transcription factor RITF1. The transgenic plants overexpressing *RSA1* or *RITF1* also display enhanced expression of *SOS1*, *At5g14130*, and *At2g36690*, further confirming that RSA1 and RITF1 are important positive regulators for *SOS1*, *At5g14130*, and *At2g36690* (Figure 6C and 6D). It is clear that the RSA1-RITF1 complex positively controls expression of *SOS1*, which encodes a plasma-membrane localized Na^+^/H^+^ antiporter (Figure 6A and 6C). Presumably, the Na^+^/H^+^ antiporter activity of SOS1 is reduced in *rsa1-1* under salt stress, leading to increased accumulation of Na^+^ in *rsa1-1* plants (Figure 1G). The SOS3-SOS2 protein kinase complex phosphorylates SOS1 and thereby activates its Na^+^/H^+^ antiporter activity [2]. Thus, the RSA1-RITF1 complex and the SOS3-SOS2 protein kinase complex regulate a common downstream target, SOS1, at two different levels in two separate cellular compartments, i.e., there is transcriptional regulation through RSA1-RITF1 in the nucleus and posttranslational modification by SOS3-SOS2 in the cytosol (Figure 7). In addition, constitutive expression of *SOS1* in the *rsa1-1* background can only partially rescue (∼12.5% improvement compared to the *rsa1-1* plants) the hypersensitive phenotype of *rsa1-1* in response to NaCl (Figure S10A and S10C). These results suggest that besides SOS1, other components in the signal transduction pathways mediated by RSA1 under salt stress are essential for salt stress tolerance.

How does RSA1 sense changes in free calcium levels elicited by salt stress in the nucleus and then transduce signals to downstream signal transduction components and eventually activate gene expression? Calcium signals are detected in the nuclei of root nodules of legume plants [20]–[22]. Similar observations have been reported for other plant species (e.g., tobacco) in response to external stimuli [23]–[27]. Although the ultimate sources of nuclear, free calcium are debated, it is now clear that a nuclear-associated change in calcium levels acts as a secondary messenger for downstream signal transduction events as evidenced in the symbiotic signaling pathway in legumes for nodule formation [28]–[30]. Following Nod factor induction of nuclear-calcium spiking in legumes, a nuclear-localized calcium- and calmodulin-dependent protein kinase (CCaMK) and nuclear-associated transcription factors and other proteins are involved in the signaling transduction pathway for nodulation [31]–[35]. RSA1 is a nuclear-localized calcium-binding protein. Thus, RSA1 may sense changes in nuclear, free Ca^2+^ and then transduce this nuclear calcium signal to regulate gene expression that helps plants to cope with salt stress. We used a yeast two-hybrid screening strategy to identify RSA1 interacting partners, and a bHLH transcription factor RITF1 was isolated in this process. RSA1 physically interacts with RITF1 both *in vitro* and *in vivo*. ChIP-qPCR analysis revealed that RITF1 is able to directly bind to *cis* promoter elements of RSA1 downstream target genes. Determining how RSA1 or the RSA1-RITF1 complex function in the nuclear calcium-sensing pathway required for gene regulation under salt stress is very challenging because tools to manipulate calcium levels in the nucleus of intact plants are lacking. In the current study, we observed that the calcium-binding EF-hand motif in RSA1 is critical for RSA1 function *in vivo* (Figure 3C and 3D). Under salt stress, RSA1 and RITF1 can transiently activate the *SOS1* promoter, and the calcium-binding EF-hand motif in RSA1 is crucial for the activation of the *SOS1* promoter as determined in a dual luciferase reporter assay (Figure 6I). Furthermore, the *rsa1-2ritf1* double mutant plants do not display any additive effect of *rsa1-2* and *ritf1* mutations under salt stress or oxidative stress (Figure S8) and overexpression of *RITF1* in the *rsa1-1* background is unable to rescue the salt hypersensitive phenotype of *rsa1-1* (Figure S10B and S10D). Together, these results suggest that RSA1 senses the salt-induced changes in nuclear free calcium and interacts with a transcription factor, RITF1, for gene expression, and that both RSA1 and RITF1 are required for salt tolerance. RITF1 may require posttranslational modifications (i.e., phosphorylation) to be active, and RSA1 is likely involved in these processes. Popescu et al. [36] reported that RITF1 is phosphorylated by multiple MAPKs in the presence of different MAPKKs in a protein microarray analysis. Further investigation is required to determine which MAPK phosphorylates RITF1 and the relationship between the RSA1-mediated calcium signal and phosphorylation of RITF1 by certain MAPK(s).

The importance of RSA1 and RITF1 proteins in salt tolerance is indicated by the loss-of-function and gain-of-function studies. The *rsa1-1* mutation causes a hypersensitive phenotype in response to NaCl. The increased sensitivity in *rsa1-1* is specific to Na^+^ (Figure 1 and Figure S1). This phenotype of *rsa1-1* is very different from that of previously described *salt overly sensitive* (*sos*) mutants (*sos1* through *sos6*), which are hypersensitive to both Na^+^ and Li^+^ [37]–[40]. In addition, as indicated by comparison with publicly available expression datasets, most of the differentially expressed genes with or without salt stress in *rsa1-1* (as revealed in the microarray analysis) are not responsive to salt stress treatments in the wild type (Figure S5 and S6). Therefore, mis-regulation of these genes in *rsa1-1* mutant plants may contribute to the overall increased salt sensitivity. Like *rsa1-1* mutant plants, *ritf1* mutant plants are more sensitive to salt stress than the wild type (Figure 5A and 5B). Overexpression of *RSA1* or *RITF1* improves the performance of transgenic *Arabidopsis* plants under salt stress (Figure 3E, 5F–5H). Furthermore, the excessive production of deleterious ROS in *rsa1-1* plants may contribute to the increased sensitivity of the *rsa1-1* mutant to salt stress (Figure 2 and 7). Upon salt stress, ROS accumulate to higher levels in the *rsa1-1* mutant than in the wild type (Figure 2), which indicates that RSA1 is required for maintenance of ROS levels. The microarray analysis showed that RSA1 controls expression of genes that encode ROS scavenging enzymes and redox-related proteins. Improper functioning of these ROS detoxifying proteins is the possible cause of over-accumulation of ROS in *rsa1-1*. SOS1 is involved in maintaining proper ROS levels because ROS over-accumulate in the *sos1-1* mutant plants under salt stress, and the *sos1-1* mutation affects expression of several ROS detoxifying genes [6]. Therefore, reduced expression of *SOS1* in *rsa1-1* may also contribute to the increased salt sensitivity of *rsa1-1*. Finally, database searches revealed that close homologs of RSA1 are present in other plant species including monocots (such as rice and maize) and dicots (such as cucumber and tomato) (Figure S11A). In all of these plant species except grape, RSA1 homologs exist as a single copy gene. In contrast, two to three RITF1 close homologs are present in *Arabidopsis* and other plant species with RSA1 homologs (Figure S11B). These findings suggest that the RSA1-RITF1 complex is conserved across plant species. It is also possible that mechanisms of RSA1 and RITF1 function under salt stress are conserved in these plant species. Because overexpression of *RSA1* or *RITF1* increases salt tolerance in *Arabidopsis*, manipulation of RSA1 and RITF1 (or their close homologs) levels in salt-sensitive crops such as rice and tomato may increase their salt tolerance.

## Materials and Methods

### Plant material and growth conditions

A firefly luciferase reporter gene driven by the stress-responsive *RD29A* promoter [41] was introduced into *Arabidopsis* plants in the Columbia *glabrous1* (*gl1*) background. Seeds from one homozygous line (referred to as the wild type) were mutagenized with ethyl methanesulfonate (EMS), and M_2_ seeds were used to screen for hypersensitive mutants in the presence of 100 mM NaCl with a modified root-bending assay [13], [14], [42]. *Arabidopsis* seedlings on Murashige and Skoog (MS) medium agar plates (1× MS salts, 2% sucrose, 1.2% agar, pH 5.7) were routinely grown in vertical orientation under cool, white light (∼120 µmol m^−2^ s^−1^) at 21°C with a 16-h-light/8-h-dark photoperiod. Soil-grown plants were kept under cool, white light (∼100 µmol m^−2^ s^−1^) with a 16-h-light/8-h-dark photoperiod at 21°C; the potting soil was a 1∶1 mixture of Metro Mix 360 and LC1 (Sun Gro Horticulture, Bellevue, WA).

Seeds of *rsa1-2* (SALK_007142), *rsa1-3* (CS16026), and *rift1* (CS811403) were obtained from the *Arabidopsis* Biological Resource Center (ABRC; Columbus, OH).

### Microarray analysis and qRT-PCR analysis

Six-d-old wild type and *rsa1-1* mutant seedlings grown on MS medium (1× MS salts, 2% sucrose, 1.2% agar, pH 5.7) were transferred to MS medium supplemented with 0 or 120 mM NaCl and allowed to grow for an additional 24 h. Total RNA was prepared as described [43]. Microarray analysis was carried out using *Arabidopsis* Affymetrix ATH1 GeneChips in the School of Medicine, University of Maryland, Baltimore as described [42], [44], [45]. The microarray data discussed in this study have been deposited in NCBI's Gene Expression Omnibus [46] and are accessible through GEO Series accession number GSE39236. All qRT-PCR experiments were performed as described [43].

### Chromatin immunoprecipitation (ChIP) assay

The *RITF1* gene including its native promoter (2.8 kb) was amplified by PCR and cloned into pEarlyGate301. The resulting construct (*pRITF1:RITF1-HA*) was then transformed into wild-type plants by a floral dip method with *Agrobacterium tumefaciens* (strain GV3101)-mediated transformation [47]. ChIP assays were carried out with 15-d-old seedlings expressing *pRITF1:RITF1-HA* that were grown on horizontally oriented MS agar plates (1× MS salts, 2% sucrose, 0.6% agarose, pH 5.7) as described [48], [49]. Briefly, seedlings were crosslinked with 1% formaldehyde, and chromatin was isolated, sonicated (Fisher Biodismembrator, model120), and pre-cleared with salmon sperm DNA/protein-A agarose beads for 1 h. Samples were then immunoprecipitated with anti-HA antibody (Sigma, H6908) at 4°C overnight. The chromatin antibody complex was precipitated with salmon sperm DNA/protein-A agarose beads, washed with four different buffers for 5 min per buffer, and reverse-crosslinked with 200 mM NaCl in elution buffer (1% SDS, 0.1 M NaHCO_3_) for 6 h at 65°C. Proteins in the complex were removed by proteinase K at 45°C for 1 h. DNA was precipitated in the presence of two volumes of ethanol, 1/10 volume of 3 M sodium acetate (pH 5.2), and 2 µg of glycogen. Real-time PCR analysis was performed with immunoprecipitated DNA using a Bio-Rad CFX96 Real-Time System.

### Electron scanning microscopy of *rsa1-1* roots

Root morphology of wild-type and *rsa1-1* seedlings with or without salt stress was observed with a scanning electron microscope (SEM, AMRAY 1820D). Seedlings were first fixed on MS medium with GA fixation solution (2% glutaraldehyde, 1× MS salts, 2% sucrose, pH 5.7) for 30 min at room temperature. Roots of fixed seedlings were then excised and placed in GA fixation solution overnight at 4°C. After roots were placed in a secondary fixation solution containing 1% OsO_4_ in distilled H_2_O (pH 7.0) for 60 min, they were sequentially washed with H_2_O (three times, 10 min each time), and then with 70, 95, and 100% ethanol (once for 10 min for each ethanol concentration). Roots were then critical point dried using CO_2_. Roots were mounted on stubs and shadowed with gold and palladium (4∶1) before viewing with the SEM.

### Genetic mapping of the *rsa1-1* locus and generation of RSA1-related constructs

For mapping of the *rsa1-1* locus, homozygous *rsa1-1* plants (in Columbia) were crossed to wild-type plants of Landsberg *erecta*. From the segregating F_2_ population, 657 homozygous *rsa1-1* mutants were selected, and DNA was extracted from each of these plants for mapping with simple sequence length polymorphism (SSLP) markers between Columbia and Landsberg *erecta*.

A 8.9-kb genomic DNA fragment of *At2g03150* including its putative promoter and 3′-termination sequence was amplified by PCR with the BAC clone T18E12 as a template and was cloned into the pMDC99 vector through Gateway technology (Invitrogen). The resulting binary vector (pRSA1:RSA1) was transformed into *rsa1-1* plants by a floral dip method with *A. tumefaciens* (strain GV3101)-mediated transformation. T_2_ seedlings were examined for sensitivity to 100 mM NaCl.

The DNA fragment containing the *RSA1* promoter was amplified by PCR with the pMDC99-RSA1 plasmid as a template and cloned into the binary vector pMDC164. The resulting binary vector (pRSA1:GUS) was transferred to wild-type (Columbia) plants, and pRSA1:GUS activity was observed as described [40].

The *RSA1* coding region was amplified by PCR and cloned into the pEarleyGate104 binary vector. The resulting construct (p35S:YFP-RSA1) was then transformed into wild-type plants through an *A. tumefaciens* (strain GV3101)-mediated transformation. The subcellular localization of p35S:YFP-RSA1 was determined in root tip cells of T_2_ seedlings subjected to 0 or 120 mM NaCl for 24 h using a Leica SP5X confocal microscope (Leica Microsystems). The p35S:YFP-RSA1 construct was also transferred to *rsa1-1* mutant plants to determine whether p35S:YFP-RSA1 is functional *in vivo* and to identify over-expressers of RSA1 in the *rsa1-1* mutant background. The RSA1 coding region without EF-hand motif (deletion of 12 consensus amino acids in the EF-hand motif) was amplified by PCR reactions and cloned into the pEarleyGate 104 binary vector. The resulting construct (p35S:YFP-RSA1[-EF]) was transformed into *rsa1-1* mutant plants through an *A. tumefaciens* (strain GV3101)-mediated transformation to determine whether the EF-hand motif in RSA1 is required for its function *in vivo*.

In a separate study, the *RSA1* gene including its native promoter was amplified by PCR and cloned into the pMDC107 binary vector. The resulting construct (pRSA1:RSA1-GFP) was transformed into tobacco plants, and the subcellular localization of pRSA1:RSA1-GFP protein in tobacco leaves was determined as described [43]. The same construct was also transferred to the *rsa1-1* mutant plants to determine whether pRSA1:RSA1-GFP is functional *in vivo*.

### Recombinant protein expression and calcium overlay assay

For expression of His-tagged RSA1 (corresponding to amino acids from 1171 to 1340) and RSA1ΔEF (deletion of 12 consensus amino acids in the EF-hand motif) proteins in *Escherichia coli* (*E. coli*), *RSA1* or *RSA1(-EF)* was amplified by PCR and cloned into pDEST17 through the Gateway technology (Invitrogen). The resulting plasmids (pDEST17-RSA1 and pDEST17-RSA1ΔEF) were transformed into *E. coli* strain Rosetta (DE3) pLysS (EMD Millipore). A 4 mL volume of overnight culture was inoculated into 400 mL of LB media containing 100 mg/L carbenicillin and 34 mg/L chloramphenicol and was incubated at 30°C until OD_600_ = 0.5. Protein expression was then induced overnight by adding IPTG to a final concentration of 0.4 mM at 28°C. Recombinant proteins were purified with affinity chromatography using a His-Trap column (GE Healthcare) according to the manufacturer's instructions.

Calcium overlay assay was performed as described [50], [51] with minor modifications. A 50 µg quantity of recombinant RSA1 or RSA1(-EF) was separated on SDS-PAGE and transferred to a polyvinylidene difluoride (PVDF) membrane (Millipore, 0.45-µm-pore size). After transfer, the membrane was washed three times for 20 min each time with washing buffer (60 mM KCl, 5 mM MgCl_2_, and 10 mM imidazole-HCl [pH 6.8]) and incubated with 2.5 µCi/mL ^45^CaCl_2_ (PerkinElmer) in washing buffer for 20 min at room temperature. The membranes were washed with distilled water for 30 min, dried, and exposed to x-ray film for 3 d at −80°C.

### Yeast two-hybrid screening, subcellular localization of RITF1, BiFC assay, Split-LUC assay, and Co-IP analysis

The yeast two-hybrid system and the *Arabidopsis* l-ACT cDNA expression library [52] were obtained from the ABRC (stock number CD4-22). The *RSA1* coding region was amplified by PCR and cloned into pAS1-CYH2 to generate RSA1-pAS1-CYH2 as bait. Yeast (*Saccharomyces cerevisiae* strain AH109) transformation and library screening were as described [53]. The yeast colonies expressing different combinations of bait and prey constructs were analyzed for the *LacZ* reporter gene with paper-lifting methods. Whatman filter paper discs (#1) were used to transfer yeast colonies from the media and were then dipped in liquid nitrogen for 10 s; the color reaction was then performed by imbibing the paper discs in a solution containing 60 mM Na_2_HPO_4_⋅7H_2_O, 40 mM NaH_2_PO_4_⋅H_2_O, 10 mM KCl, 1 mM MgSO_4_, 38.6 mM β-mercaptomethanol, and 0.334 mg/mL of 5-bromo-4-chloro-3-indolyl β-D-galactopyranoside (X-Gal) at 30°C.

For validation of the results obtained from the above yeast two-hybrid screening, *RSA1* and *RITF1*(*At3g06590*) coding regions were amplified by PCR and cloned into pDEST32 (pDEST32-RSA1) as bait and pDEST22 (pDEST22-RITF1) as prey, using the ProQuest™ Two-Hybrid System with Gateway Technology (Invitrogen). For potential interactions between RSA1 and RITF1 homologs (At3g17100, At3g05800, and At1g09250), coding sequences of RITF1 homologs were amplified by PCR and cloned into pDEST22. Yeast transformation and downstream analysis were performed according to the manufacturer's instructions.

The *RITF1* coding region was amplified by PCR and the PCR product was cloned into the pMDC83 vector. The resulting construct (p35S:RITF1-GFP) was then transformed into *Arabidopsis* protoplasts prepared from 4-week-old *Arabidopsis* leaves. GFP signals in transformed protoplasts were then detected with a Leica SP5X confocal microscope (Leica Microsystems). The p35S:RITF1-GFP was also transformed into *Arabidopsis* wild-type and *rsa1-1* plants to identify transgenic plants overexpressing *RITF1* in wild-type or *rsa1-1* background.

Bimolecular fluorescence complementation (BiFC) assays were conducted as described [54]. Briefly, coding regions of *RSA1* and *RITF1* were amplified by PCR and cloned into pUC-SPYNE and pUC-SPYCE, respectively. The resulting constructs (RSA1-nYFP and RITF1-cYFP) were co-transfected into protoplasts prepared from 4-week-old *Arabidopsis* leaves. YFP signals in transformed protoplasts were then detected by confocal microscopy. The RSA1-nYFP and RITF1-cYFP constructs were also transformed into *A. tumefaciens* strain C58C1 and co-infiltrated with *35S:p19* (p19 is a RNA silencing repressor protein from tomato bushy stunt virus [55]) in *A. tumefaciens* strain C58C1 into the 3-week-old leaves of tobacco (*Nicotiana benthamiana*) plants. The infiltrated tobacco plants were grown for an additional 3 d in a growth chamber under a 16-h-light/8-h-dark photoperiod at 21°C. YFP signals in transformed tobacco leaves were then detected by confocal microscopy.

For the Split-LUC assay, coding regions of RSA1 and RITF1 were amplified by PCR and cloned into the Gateway compatible firefly luciferase complementation imaging vectors (modified from original plasmids described by [56]). The resulting constructs (RSA1-nLUC and RITF1-cLUC) were transformed into *A. tumefaciens* strain C58C1 and co-infiltrated with *35S:p19* in *A. tumefaciens* strain C58C1 into the 3-week-old leaves of tobacco plants. The infiltrated tobacco plants were grown for an additional 3 d in a growth chamber under a 16-h-light/8-h-dark photoperiod at 21°C. Luciferase expression was observed with a CCD (charge-coupled device) camera as described [41].

Co-IP analysis was performed as described [57], [58] with minor modifications. Briefly, coding regions of *RSA1* and *RITF1* were amplified by PCR and cloned into pEarleyGate 202 and pEarleyGate 201, respectively. The resulting constructs (FLAG-RSA1 and HA-RITF1) were transformed into *A. tumefaciens* strain C58C1 and co-infiltrated with *35S:p19* in *A. tumefaciens* strain C58C1 into the 3-week-old leaves of tobacco (*Nicotiana benthamiana*) plants. The infiltrated tobacco plants were grown for an additional 3 d in a growth chamber under a 16-h-light/8-h-dark photoperiod at 21°C. Proteins were extracted from leaf samples with extraction buffer (50 mM Tris-HCl, pH 8.0, 150 mM NaCl, 2 mM EDTA, 2 mM DTT, 10% glycerol, 1% Triton X-100, 1 mM PMSF, 1× Halt protease inhibitor cocktail [Fisher Scientific]). The protein extracts were incubated with anti-HA antibody (Sigma; Cat. No. H6908) overnight. The immunocomplexes were collected by adding protein A agarose beads and were washed with immunoprecipitation buffer (50 mM Tris-HCl, pH 8.0, 150 mM NaCl, 2 mM EDTA, 2 mM DTT, 10% glycerol, 0.15% Triton X-100, 1 mM PMSF, 1× Halt protease inhibitor cocktail [Fisher Scientific]). The pellet (immunocomplexes with beads) was resuspended in 2× SDS-PAGE loading buffer. Eluted proteins were analyzed by immunoblotting using anti-FLAG antibody (Sigma; Cat. No. F1804) or anti-HA antibody (Sigma; Cat. No. H6908). Chemiluminescence signal was detected by autoradiography.

### Dual luciferase reporter assays

The putative *SOS1* promoter fragment (∼1.9 kb upstream of the translation start site) containing the *cis* element CACTTG that is recognized by RITF1 (as determined by the ChIP-qPCR analysis) was amplified by PCR with BAC clone F14H20 as a template. The PCR product was cloned into the transient expression vector pGreenII 0800-LUC to serve as a reporter plasmid. The coding regions of *RSA1* and *RITF1* were amplified by PCR and cloned into the transient expression vector pGreenII 62-SK to serve as effect plasmids. The *RSA1* coding region without EF-hand motif (deletion of 12 consensus amino acids in the EF-hand motif) was amplified by PCR with p35S:YFP-RSA1(-EF) as a template and cloned into the transient expression vector pGreenII 62-SK to serve as effect plasmid. The resulting plasmids (SOS1∶LUC, RSA1, RITF1, and RSA1[-EF]) were transformed into *A. tumefaciens* (strain GV3101). Four-week-old tobacco leaves were co-infiltrated with the *A. tumefaciens* (strain GV3101) harboring the above plasmids in the following combination and ratios: SOS1∶LUC (100%); SOS1∶LUC+RITF1 (1∶9); SOS1∶LUC+RITF1+RSA1 (1∶4.5∶4.5); and SOS1∶LUC+RITF1+RSA1(-EF) (1∶4.5∶4.5). The infiltrated tobacco plants were grown for an additional 3 d in a growth chamber under a 16-h-light/8-h-dark photoperiod at 21°C and were subjected to 0 or 150 mM NaCl for 24 h. Activities of firefly luciferase under the control of the *SOS1* promoter (*SOS1:LUC*) and Renillia luciferase under the control of the 35S promoter (*35S:LUC*) (both the *SOS1:LUC* and *35S:LUC* transgenes are present on the *SOS1:LUC* reporter plasmid, and *35S:LUC* served as an internal control) were measured using the reagents contained in the Dual-Luciferase Reporter Assay System (Promega) with the Modulus Microplate Multimode Reader (Turner BioSystem) as described [59]. Normalized data (ratio of luminescent signal intensity from the *SOS1:LUC* reporter and luminescent signal intensity from the internal control reporter, *35S:LUC*) from 12 independent biological samples are presented.

### Overexpression of *SOS1* in *rsa1-1*

The coding region of *SOS1* was amplified by PCR, and the PCR product was cloned into the binary vector pEarleyGate201. The resulting construct (p35S:HA-SOS1, abbreviated as SOS1 OE) was transformed into the *rsa1-1* mutant plants through an *A. tumefaciens* (strain GV3101)-mediated transformation.

### Ion content determination

Ion content in *rsa1-1* was measured as described previously [60] with minor modifications. Briefly, 1-month-old wild-type and *rsa1-1* plants grown in soil side by side in the same pots were treated with 150 mM NaCl and allowed to grow for one additional week. Shoots were harvested, dried in an oven at 65°C for 72 h, and weighed. A minimum of 2 g of dried shoot tissue was ashed at 480°C for 16 h before 2 mL of concentrated HNO_3_ was added to the ashes. After the ashes were dried on a hot plate, 10 mL of 3 M HCl was added, and the mixture was allowed to reflux for 2 h. The digests were passed through Whatman #40 filter paper, and the filtrate volumes were brought to 25 mL with 0.1 M HCI. Levels of Na^+^ and K^+^ were then determined with an inductively coupled plasma optical emission spectrometer (ICP-OES) (PerkinElmer Optima 4300 DV).

### Determination of reactive oxygen species levels

Five-d-old seedlings grown on vertically oriented MS medium (1× MS salts, 2% sucrose, 1.2% agar, pH 5.7) were transferred to MS medium supplemented with 0 or 50 mM NaCl and allowed to grow for an additional 12 h. Seedlings were incubated with 5-(and 6)-chloromethyl-2′7′-dichlorodihydrofluorescein diacetate acetyl ester (CM-H_2_DCFDA) for 30 min and washed with distilled H_2_O to remove excess CM-H_2_DCFDA. Fluorescence images were obtained with a Leica SPX5 confocal microscope (Leica Microsystems), and ROS levels were quantified based on the intensity of fluorescence with the ImageJ software (NIH, http://rsbweb.nih.gov/ij/).

For quantification of H_2_O_2_, 5-d-old wild-type and *rsa1-1* seedlings were transferred to MS medium containing 0 or 75 mM NaCl and allowed to grow for an additional 12 h. Seedlings were then harvested, and H_2_O_2_ content was determined with an Amplex red hydrogen/peroxidase assay kit (Invitrogen) according to the manufacturer's instructions.

### Determination of chlorophyll and anthocyanin accumulation

Chlorophyll was determined as described [61] with minor modifications. Chlorophyll was extracted by incubating ground seedlings in 80% acetone overnight at 4°C in darkness and with continuous shaking. The contents of chlorophyll *a* and *b* were calculated as 7.49A_664.9_+20.3A_648.2_. Anthocyanin accumulation was determined as described [62]. Briefly, anthocyanin was extracted by incubating ground seedlings in acidified methanol (1% HCl) overnight at 4°C in darkness and with continuous shaking. The amount of anthocyanin was calculated as A_530_ - 0.33A_657_.

## Supporting Information

Figure S1 Root morphology of wild-type and *rsa1-1* seedlings under salt stress, and responses of wild-type and *rsa1-1* seedlings to LiCl, CsCl, or mannitol. (A) Morphology of wild-type and *rsa1-1* roots observed under scanning electron microscope (SEM) with or without salt stress. Five-d-old wild-type and *rsa1-1* seedlings grown on MS medium were transferred to MS medium containing 0 or 100 mM NaCl and allowed to grow for an additional 5 d. (B)–(D) Responses of wild-type and *rsa1-1* seedlings to LiCl, CsCl, or mannitol. Five-d-old wild-type and *rsa1-1* seedlings grown on MS medium were transferred to MS medium supplemented with different levels of LiCl (B), CsCl (C) or mannitol (D) and allowed to grow for an additional 8 d. Root elongation or shoot fresh weight was measured and is shown as a percentage relative to growth on normal MS medium. WT, wild type. Error bars represent the standard deviation (n = 30–40). The experiments in Figure S1 were performed at least three times with similar results, and data from one representative experiment are presented.
(TIF)

Figure S2 Map-based cloning of *RSA1* and genetic complementation of *rsa1-1*. (A) Map-based cloning of *RSA1*. (a) Numbers of recombination are from 657 F_2_ progeny seedlings that are homozygous for *rsa1-1* phenotypes. (b) Structure of the *RSA1* gene and positions of *rsa1-1*, *rsa1-2* (T-DNA), and *rsa1-3* (T-DNA) mutations are indicated. Filled boxes indicate exons, and lines between boxes indicate introns. (c) Functional motif on deduced RSA1 polypeptide. (B) Gene complementation of the *rsa1-1* mutant by the wild-type *RSA1* gene, and growth responses of *rsa1-2* and *rsa1-3* seedlings to 100 mM NaCl. Five-d-old of seedlings grown on MS medium were transferred to MS medium containing 0 or 100 mM NaCl and allowed to grow for an additional 14 d. (C) Quantification of root growth of plants related to gene complementation analysis shown in (B). (D) Expression of *RSA1* in wild-type, *rsa1-2*, and *rsa1-3* seedlings. The qRT-PCR analysis was carried out with 14-d-old wild-type, *rsa1-2*, and *rsa1-3* seedlings grown on MS medium. (E) Quantification of shoot fresh weight of wild-type, *rsa1-2*, and *rsa1-3* plants shown in (B). Error bars indicate the standard deviation (n = 40 in [C], 4 in [D], and 15 in [E]). WT, wild type. The experiments in Figure S2 except for Figure S2 (A) were performed at least three times with similar results, and data from one representative experiment are presented.
(TIF)

Figure S3 Expression of *RSA1* in different tissues; complementation of *rsa1-1* by the *p35S:YFP-RSA1* transgene or by the *RSA1:RSA1-GFP* transgene; and alignment of the EF-hand motif in *Arabidopsis*. (A) *pRSA1:GUS* expression in seedlings and various tissues of wild-type plants. (B) Transcript levels of *RSA1* in various tissues of wild-type plants as determined by qRT-PCR analysis. (C) Gene complementation of *rsa1-1* by the *p35S:YFP-RSA1* transgene as indicated by root elongation. (D) Gene complementation of *rsa1-1* by the *RSA1:RSA1-GFP* transgene as indicated by root elongation. In (C) and (D), 5-d-old seedlings grown on MS medium were transferred to MS medium containing different levels of NaCl and allowed to grow for an additional 7 d. (E) Comparison of core consensus amino acid sequences of the EF-hand motif in RSA1 with those of other EF-hand motif containing proteins in *Arabidopsis*. Alignment was performed with ClastalW program as a part of the Bioedit package (version 7.09) with default settings ([63]; http://www.mbio.ncsu.edu/BioEdit/bioedit.html). Identical or conserved amino acid residues are shaded in black or grey, respectively. (F) *RSA1* expression in wild-type and *rsa1-1* plants expressing *p35S:YFP-RSA1* as determined by qRT-PCR analysis. qRT-PCR analysis in (B) and (F) was carried out with total RNA isolated from 14-d-old seedlings grown on MS medium. Error bars represent the standard deviation (n = 4 in [B] and [F], and 40 in [C]–[D]). The experiments in Figure S3 were performed at least three times with similar results, and data from one representative experiment are presented.
(TIF)

Figure S4 RSA1 interacts with RITF1, and *RITF1* expression in *ritf1* mutant plants and transgenic plants expressing *p35S:RITF1*. (A) RSA1 interacts with RITF1 *in vivo* as determined by BiFC assays in *Arabidopsis* protoplasts. YFP images were detected at an approximate frequency of 4.05% (44 out of 1,086 protoplasts analyzed exhibited BiFC events). (B) *RITF1* expression in wild-type and *ritf1* mutant plants as determined by qRT-PCR analysis. (C) *RITF1* expression in wild-type and transgenic plants expressing *p35S:RITF1* as determined by qRT-PCR analysis. qRT-PCR analysis in (B) and (C) was carried out with total RNA isolated from 14-d-old seedlings grown on MS medium. Error bars represent the standard deviation (n = 4). The experiments in Figure S4 were performed at least four times with similar results, and data from one representative experiment are presented.
(TIF)

Figure S5 Hierarchical clustering analysis of genes in wild-type plants in response to salt stress treatments using publicly available gene expression data; these genes showed increased (A) and reduced (B) expression patterns in *rsa1-1* under control conditions in our microarray analysis. Hierarchical clustering analysis was performed in Genevestigator with Hierarchical Clustering Tool (https://www.genevestigator.com/gv/user/gvLogin.jsp) [64]. Scale bars at the top indicate the relative expression level (green, repression; red, induction) of a gene compared to the non-stressed condition in wild-type plants.
(TIF)

Figure S6 Hierarchical clustering analysis of genes in wild type plants in response to salt stress treatments using publicly available gene expression data; these genes showed increased (A) and reduced (B) expression patterns in *rsa1-1* under salt stress in our microarray analysis. Hierarchical clustering analysis was performed in Genevestigator with Hierarchical Clustering Tool (https://www.genevestigator.com/gv/user/gvLogin.jsp) [64]. Scale bars at the top indicate the relative expression level (green, repression; red, induction) of a gene compared to the non-stressed condition in wild-type plants.
(TIF)

Figure S7 Validation of microarray results by qRT-PCR analysis and ChIP-qPCR analysis of the *At2g39040* gene. (A)–(D) Validation of microarray results by qRT-PCR. Six-d-old wild-type and *rsa1-1* seedlings grown on MS medium were transferred to MS medium containing 0 or 120 mM NaCl and allowed to grow for an additional 24 h. WT, wild type. *At2g39040*, *At1g10480*, *At3g56980*, and *At5g67400* encode peroxidase, zinc finger protein 5, basic helix-loop-helix (bHLH) DNA-binding superfamily protein, and root hair specific 19 with putative peroxidase activity, respectively. (E) *RITF1* expression in wild-type and transgenic plants expressing *RITF1:RITF1-HA* as determined by qRT-PCR analysis. Total RNA was isolated from 14-d-old seedlings grown on MS medium. (F) ChIP-qPCR analysis of three areas of the *At2g39040* gene. Regions of amplification: A (containing two copies of core *cis* element CATATG at two different sites) = −833 to −588; B (containing two core *cis* elements CAATTG and CAAGTG) = −143 to +6; C (containing no core *cis* element; serving as negative control) = +171 to +269 base pairs relative to the translation start site. Error bars indicate the standard deviation (n = 4). The experiments in Figure S7 were performed at least three times with similar results, and data from one representative experiment are presented.
(TIF)

Figure S8 Responses of wild-type, *ritf1*, *rsa1-2*, and *rsa1-2ritf1* seedlings to salt stress and oxidative stress. (A) Seed germination of wild type, *ritf1*, *rsa1-2*, and *rsa1-2ritf1* double mutant under 75 mM NaCl treatment. There were 80–150 seeds per genotype per biological replicate. Seeds in which the radical had emerged through the seed coat were considered germinated. (B) Growth responses of wild-type, *ritf1*, *rsa1-2*, and *rsa1-2ritf1* seedlings to H_2_O_2_. (C) Fresh weight of wild-type, *ritf1*, *rsa1-2*, and *rsa1-2ritf1* seedlings under H_2_O_2_ treatment. In (B) and (C), seeds were sown directly on MS medium supplemented with various levels of H_2_O_2_ and allowed to grown for an additional 10 d. WT, wild type. One-way ANOVA (Tukey-Kramer test) was performed, and statistically significant differences are indicated by different lowercase letters (p<0.01). Error bars represent the standard deviation (n = 30–40). The experiments in Figure S8 were performed at least three times with similar results, and data from one representative experiment are presented.
(TIF)

Figure S9 Yeast two-hybrid analysis of RSA1 and RITF1 homologs, and growth responses of the T-DNA mutant plants of RITF1 homologs under salt stress. (A) RSA1 does not interact with three close homologs of RITF1 as determined by yeast two-hybrid analysis. Yeast strain MaV203 co-transformed with different combinations of bait and prey was subjected to x-gal assay. Yeast cells grown on SD medium-L-W or SD medium-L-W-H+3-AT are shown. 3-AT, 3-amino-1,2,4-triazole. L, W, H, symbols for amino acids leucine, tryptophan, and histidine, respectively. SD, yeast minimal media. (B) Expression of *At3g05800*, *At1g09250*, or *At3g17100* in the corresponding T-DNA mutant plants. *GK233G09*, *GK428G06*, and *SALK_022587* are the T-DNA mutants of *At3g05800*, *At1g09250*, and *At3g17100*, respectively. qRT-PCR analysis was carried out with total RNA isolated from 14-d-old seedlings grown on MS medium. (C) Morphology of T-DNA mutant plants of RITF1 homologs. (D) Growth responses of T-DNA mutants of RITF1 homologs to salt stress. Five-d-old seedlings grown on MS medium were transferred to MS medium containing 0 or 120 mM NaCl and allowed to grow for an additional 7 d. (E) Shoot fresh weight of plants shown in (D). WT, wild type. Error bars represent the standard deviation (n = 4 in [B] and 40 in [E]). The experiments in Figure S9 were performed at least three times with similar results, and data from one representative experiment are presented.
(TIF)

Figure S10 Phenotypes of *rsa1-1* transgenic plants expressing *p35S:SOS1* or *p35S:RITF1*. Five-d-old seedlings grown on MS medium were transferred to MS medium supplemented with various levels of NaCl and allowed to grow for an additional 10 d (for [C] and [D]). (A) *SOS1* expression in wild-type and *rsa1-1* plants expressing *p35S:SOS1*. (B) *RITF1* expression in wild-type and *rsa1-1* plants expressing *p35S:RITF1*. qRT-PCR analysis in (A) and (B) was performed with total RNA isolated from 14-d-old seedlings grown on MS medium. (C) Root elongation of wild-type, *rsa1-1*, and one representative line of *rsa1-1* plants expressing *p35S:SOS1* in response to 0 or 100 mM NaCl. (D) Root elongation of wild-type, *rsa1-1*, and one representative line of *rsa1-1* plants expressing *p35S:RITF1* in response to 0 or 100 mM NaCl. One-way ANOVA (Tukey-Kramer test) was performed, and statistically significant differences are indicated by different lowercase letters (p<0.01). Error bars represent the standard deviation (n = 4 in [A] and [B], 30–40 in [C] and [D]). The experiments in Figure S10 were performed at least three times with similar results, and data from one representative experiment are presented.
(TIF)

Figure S11 Comparison of AtRSA1 and AtRITF1 with their close homologs in other plant species. The phylogenetic tree was generated with the Phylogeny.fr platform (http://www.phylogeny.fr/version2_cgi/advanced.cgi) as described [65]. Scale bar indicates branch length. (A) Phylogenetic tree of AtRSA1 and its close homologs. The protein identities are as follow: AtRSA1 (*Arabidopsis thaliana*, NP_178414), ZmRSA1 (*Zea mays*, AFW76494), OsRSA1_Indica (*Oryza sativa* Indica Group, EEC80815), OsRSA1_Japonica (*Oryza sativa* Japonica Group, EEE65891), CrRSA1 (*Capsella rubella*, EOA23219), PtRSA1(*Populus trichocarpa*, XP_002316125), VvRSA1.1 (*Vitis vinifera*, CBI31934), VvRSA1.2 (*Vitis vinifera*, XP_002268851), SlRSA1 (*Solanum lycopersicum*, XP_004236885), GmRSA1(*Glycine max*, XP_003520085), and CsRSA1 (*Cucumis sativus*, XP_004143774). (B) Phylogenetic tree of AtRITF1 and its close homologs. The protein identities are as follow: AtRITF1 (*Arabidopsis thaliana*, NP_566287), At3g05800 (*Arabidopsis thaliana*, NP_566260), At1g09250 (*Arabidopsis thaliana*, NP_563839), At3g17100 (*Arabidopsis thaliana*, NP_566567), CsRITF1.1 (*Cucumis sativus*, XP_004148798), CsRITF1.2 (*Cucumis sativus*, XP_004135755), GmRITF1.1 (*Glycine max*, XP_003550916), GmRITF1.2 (*Glycine max*, XP_003520791), GmRITF1.3 (*Glycine max*, XP_003536039), SlRITF1.1 (*Solanum lycopersicum*, XP_004229017), SlRITF1.2 (*Solanum lycopersicum*, XP_004230318), VvRITF1.1 (*Vitis vinifera*, XP_002279307), VvRITF1.2 (*Vitis vinifera*, XP_002281846), VvRITF1.3 (*Vitis vinifera*, XP_002270621), PtRITF1.1 (*Populus trichocarpa*, XP_002312320), PtRITF1.2 (*Populus trichocarpa*, XP_002330080), PtRITF1.3 (*Populus trichocarpa*, XP_002325095),), CrRITF1.1 (*Capsella rubella*, EOA31396), CrRITF1.2 (*Capsella rubella*, EOA31360), CrRITF1.3 (*Capsella rubella*, EOA29635), OsRITF1.1 (*Oryza sativa Indica*, EAY89737), OsRITF1.2 (*Oryza sativa* Japonica, BAC21355), OsRITF1.3 (*Oryza sativa* Japonica, NP_001172473), ZmRITF1.1 (*Zea mays*, NP_001142628), ZmRITF1.2 (*Zea mays*, AFW80198), ZmRITF1.3 (*Zea mays*, NP_001144220), and ZmRITF1.4 (*Zea mays*, DAA64215).
(TIF)

Table S1 Genetic analysis of the *rsa1-1* mutant (wild type [female]×*rsa1-1* [male] cross).
(PDF)

Table S2 Differentially expressed genes in *rsa1-1* under the control condition as determined by microarray analysis. (A) Genes with increased expression in *rsa1-1* without stress as determined by microarray analysis. (B) Genes with reduced expression in *rsa1-1* without stress as determined by microarray analysis.
(PDF)

Table S3 Differentially expressed genes in *rsa1-1* under salt stress as determined by microarray analysis. (A) Genes with increased expression in *rsa1-1* under salt stress as determined by microarray analysis. (B) Genes with reduced expression in *rsa1-1* under salt stress as determined by microarray analysis.
(PDF)

Table S4 Genes that are differentially expressed in *rsa1-1* under both control and salt stress conditions as determined by microarray analysis. (A) Genes with increased expression in *rsa1-1* under both control and salt stress conditions as determined by microarray analysis. (B) Genes with reduced expression in *rsa1-1* under both control and salt stress conditions as determined by microarray analysis.
(PDF)

Table S5 Putative binding sites of bHLH transcription factors (TFs) in promoters of genes differentially expressed in *rsa1-1* with or without salt stress as revealed by microarray analysis. (A) Putative bHLH TFs binding sites in promoters of genes with increased expression in *rsa1-1* without stress. (B) Putative bHLH TFs binding sites in promoters of genes with reduced expression in *rsa1-1* without stress. (C) Putative bHLH TFs binding sites in promoters of genes with increased expression in *rsa1-1* under salt stress. (D) Putative bHLH TFs binding sites in promoters of genes with reduced expression in *rsa1-1* under salt stress.
(PDF)

Table S6 Primers used in this study. (A) Primers used for ChIP-qPCR in this study. (B) Remaining primers used in this study.
(PDF)
