# Supplementary figures and images for: A Nuclear Calcium-Sensing Pathway Is Critical for Gene Regulation and Salt Stress Tolerance in Arabidopsis
Source: PLoS Genet. 2013 Aug 29;9(8):e1003755. doi: 10.1371/journal.pgen.1003755 (PMC3757082; doi:10.1371/journal.pgen.1003755)

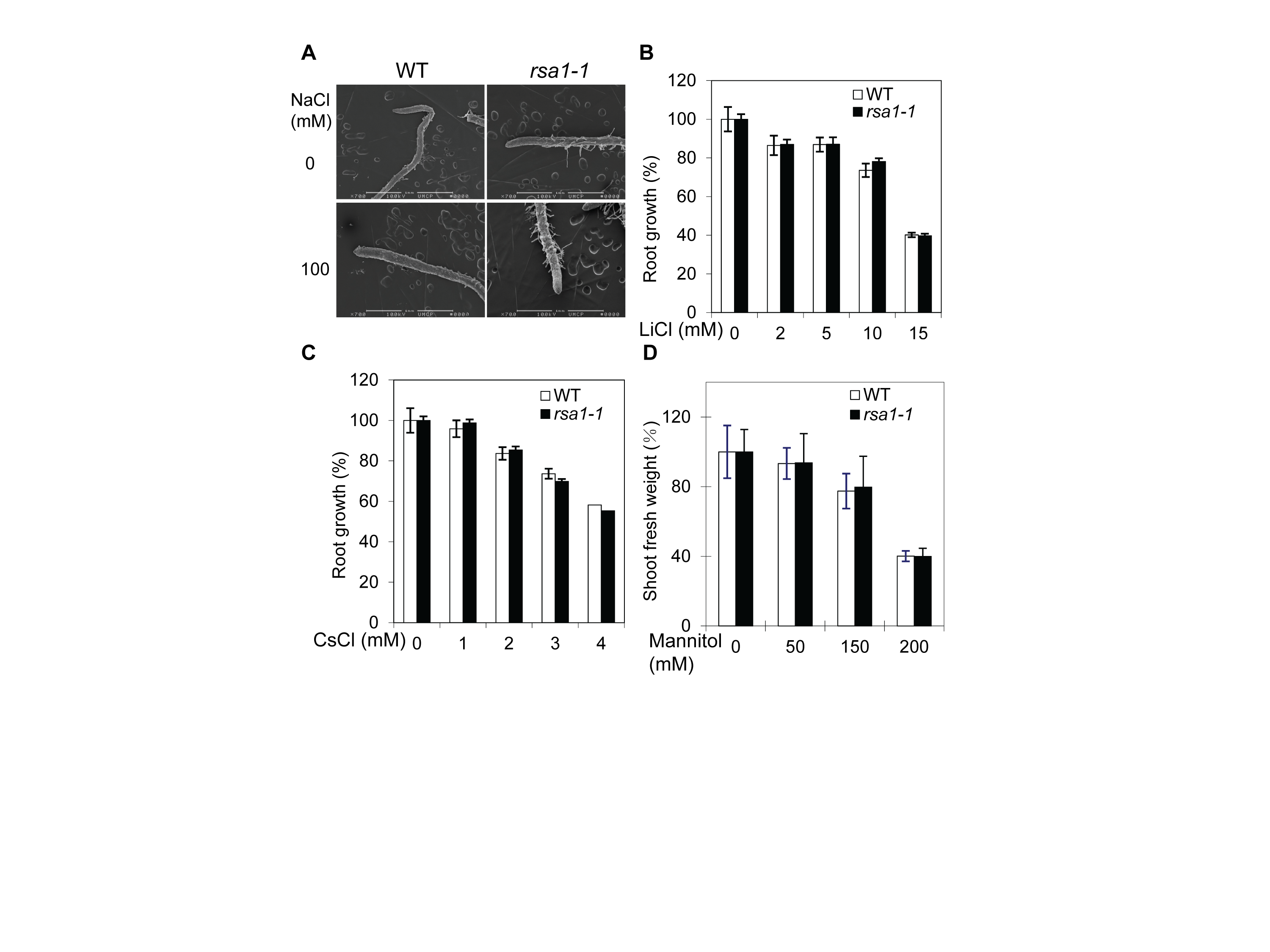

Supplement: Figure S1 — Root morphology of wild-type and rsa1-1 seedlings under salt stress, and responses of wild-type and rsa1-1 seedlings to LiCl, CsCl, or mannitol. (A) Morphology of wild-type and rsa1-1 roots observed under scanning electron microscope (SEM) with or without salt stress. Five-d-old wild-type and rsa1-1 seedlings grown on MS medium were transferred to MS medium containing 0 or 100 mM NaCl and allowed to grow for an additional 5 d. (B)–(D) Responses of wild-type and rsa1-1 seedlings to LiCl, CsCl, or mannitol. Five-d-old wild-type and rsa1-1 seedlings grown on MS medium were transferred to MS medium supplemented with different levels of LiCl (B), CsCl (C) or mannitol (D) and allowed to grow for an additional 8 d. Root elongation or shoot fresh weight was measured and is shown as a percentage relative to growth on normal MS medium. WT, wild type. Error bars represent the standard deviation (n = 30–40). The experiments in Figure S1 were performed at least three times with similar results, and data from one representative experiment are presented. (TIF) [file pgen.1003755.s001.tif]

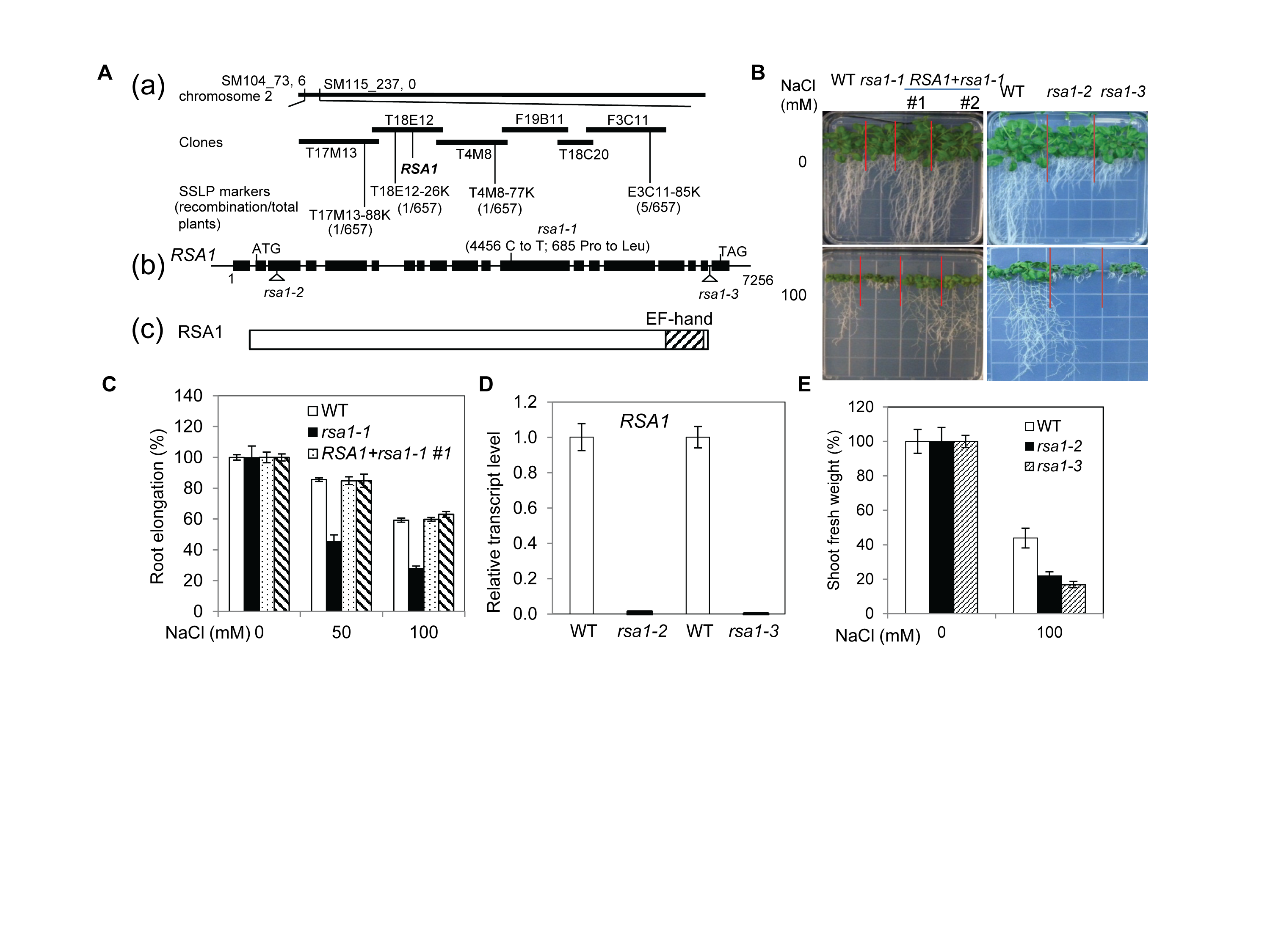

Supplement: Figure S2 — Map-based cloning of RSA1 and genetic complementation of rsa1-1. (A) Map-based cloning of RSA1. (a) Numbers of recombination are from 657 F2 progeny seedlings that are homozygous for rsa1-1 phenotypes. (b) Structure of the RSA1 gene and positions of rsa1-1, rsa1-2 (T-DNA), and rsa1-3 (T-DNA) mutations are indicated. Filled boxes indicate exons, and lines between boxes indicate introns. (c) Functional motif on deduced RSA1 polypeptide. (B) Gene complementation of the rsa1-1 mutant by the wild-type RSA1 gene, and growth responses of rsa1-2 and rsa1-3 seedlings to 100 mM NaCl. Five-d-old of seedlings grown on MS medium were transferred to MS medium containing 0 or 100 mM NaCl and allowed to grow for an additional 14 d. (C) Quantification of root growth of plants related to gene complementation analysis shown in (B). (D) Expression of RSA1 in wild-type, rsa1-2, and rsa1-3 seedlings. The qRT-PCR analysis was carried out with 14-d-old wild-type, rsa1-2, and rsa1-3 seedlings grown on MS medium. (E) Quantification of shoot fresh weight of wild-type, rsa1-2, and rsa1-3 plants shown in (B). Error bars indicate the standard deviation (n = 40 in [C], 4 in [D], and 15 in [E]). WT, wild type. The experiments in Figure S2 except for Figure S2 (A) were performed at least three times with similar results, and data from one representative experiment are presented. (TIF) [file pgen.1003755.s002.tif]

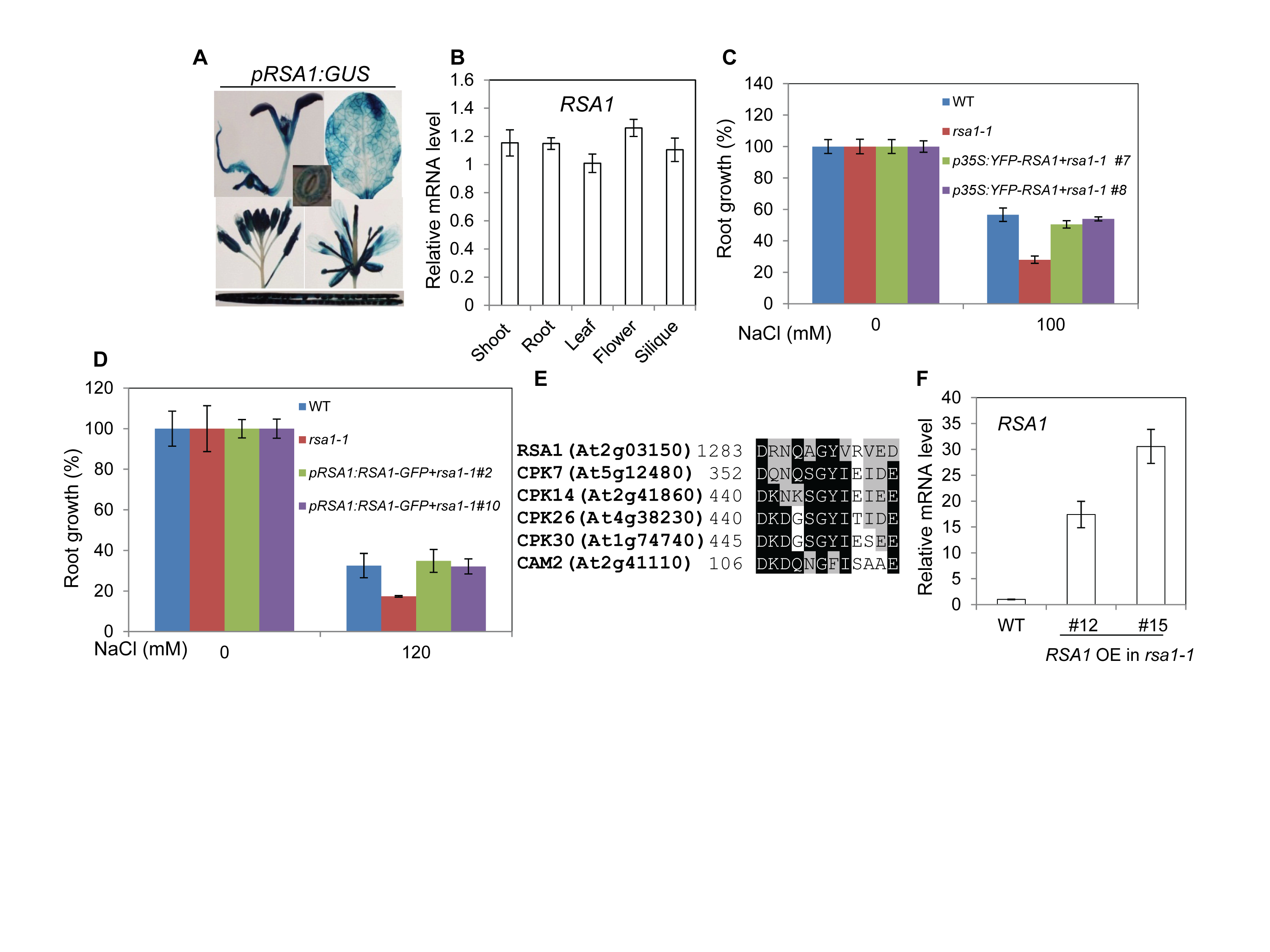

Supplement: Figure S3 — Expression of RSA1 in different tissues; complementation of rsa1-1 by the p35S:YFP-RSA1 transgene or by the RSA1:RSA1-GFP transgene; and alignment of the EF-hand motif in Arabidopsis. (A) pRSA1:GUS expression in seedlings and various tissues of wild-type plants. (B) Transcript levels of RSA1 in various tissues of wild-type plants as determined by qRT-PCR analysis. (C) Gene complementation of rsa1-1 by the p35S:YFP-RSA1 transgene as indicated by root elongation. (D) Gene complementation of rsa1-1 by the RSA1:RSA1-GFP transgene as indicated by root elongation. In (C) and (D), 5-d-old seedlings grown on MS medium were transferred to MS medium containing different levels of NaCl and allowed to grow for an additional 7 d. (E) Comparison of core consensus amino acid sequences of the EF-hand motif in RSA1 with those of other EF-hand motif containing proteins in Arabidopsis. Alignment was performed with ClastalW program as a part of the Bioedit package (version 7.09) with default settings ([63]; http://www.mbio.ncsu.edu/BioEdit/bioedit.html). Identical or conserved amino acid residues are shaded in black or grey, respectively. (F) RSA1 expression in wild-type and rsa1-1 plants expressing p35S:YFP-RSA1 as determined by qRT-PCR analysis. qRT-PCR analysis in (B) and (F) was carried out with total RNA isolated from 14-d-old seedlings grown on MS medium. Error bars represent the standard deviation (n = 4 in [B] and [F], and 40 in [C]–[D]). The experiments in Figure S3 were performed at least three times with similar results, and data from one representative experiment are presented. (TIF) [file pgen.1003755.s003.tif]

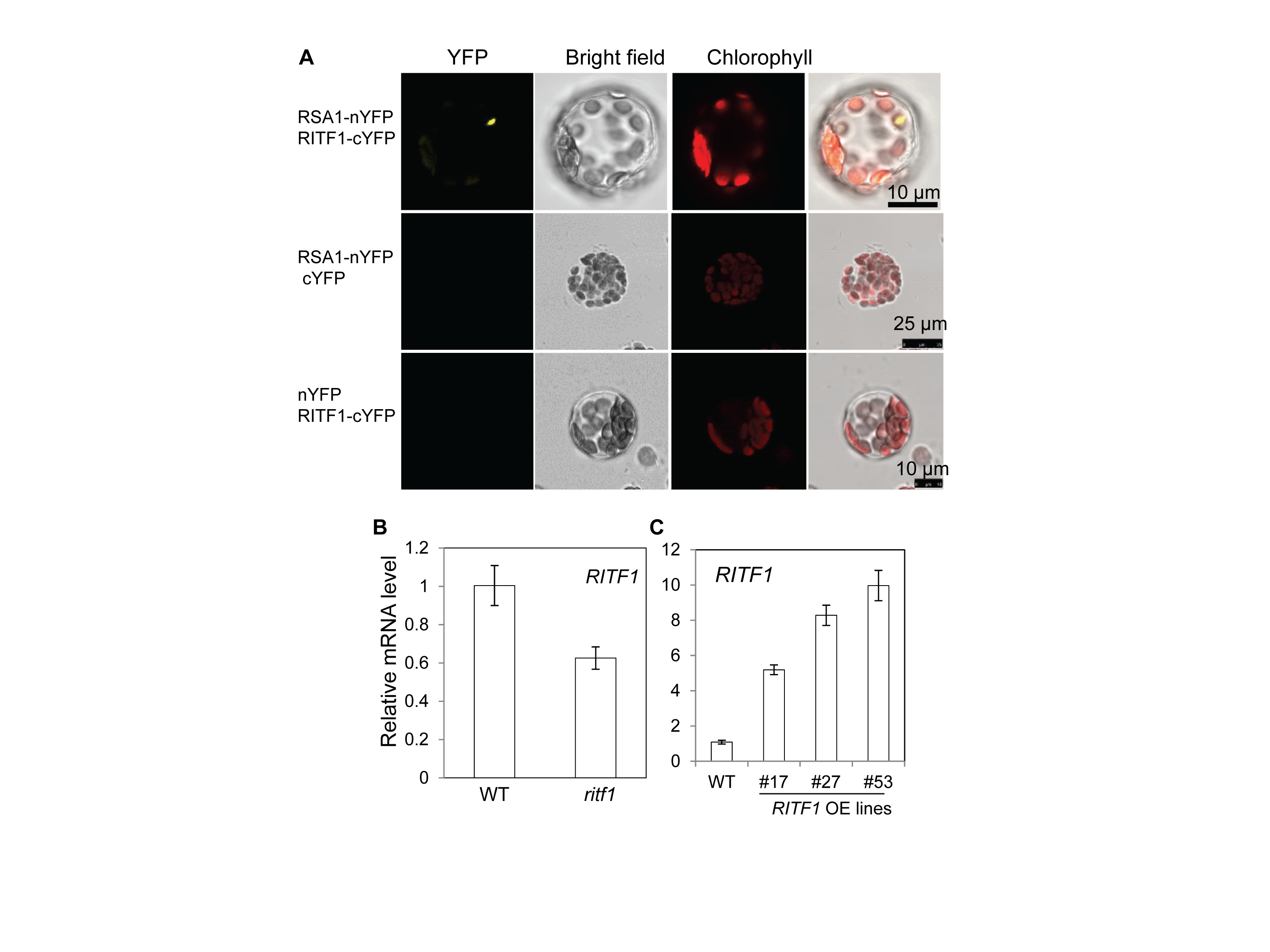

Supplement: Figure S4 — RSA1 interacts with RITF1, and RITF1 expression in ritf1 mutant plants and transgenic plants expressing p35S:RITF1. (A) RSA1 interacts with RITF1 in vivo as determined by BiFC assays in Arabidopsis protoplasts. YFP images were detected at an approximate frequency of 4.05% (44 out of 1,086 protoplasts analyzed exhibited BiFC events). (B) RITF1 expression in wild-type and ritf1 mutant plants as determined by qRT-PCR analysis. (C) RITF1 expression in wild-type and transgenic plants expressing p35S:RITF1 as determined by qRT-PCR analysis. qRT-PCR analysis in (B) and (C) was carried out with total RNA isolated from 14-d-old seedlings grown on MS medium. Error bars represent the standard deviation (n = 4). The experiments in Figure S4 were performed at least four times with similar results, and data from one representative experiment are presented. (TIF) [file pgen.1003755.s004.tif]

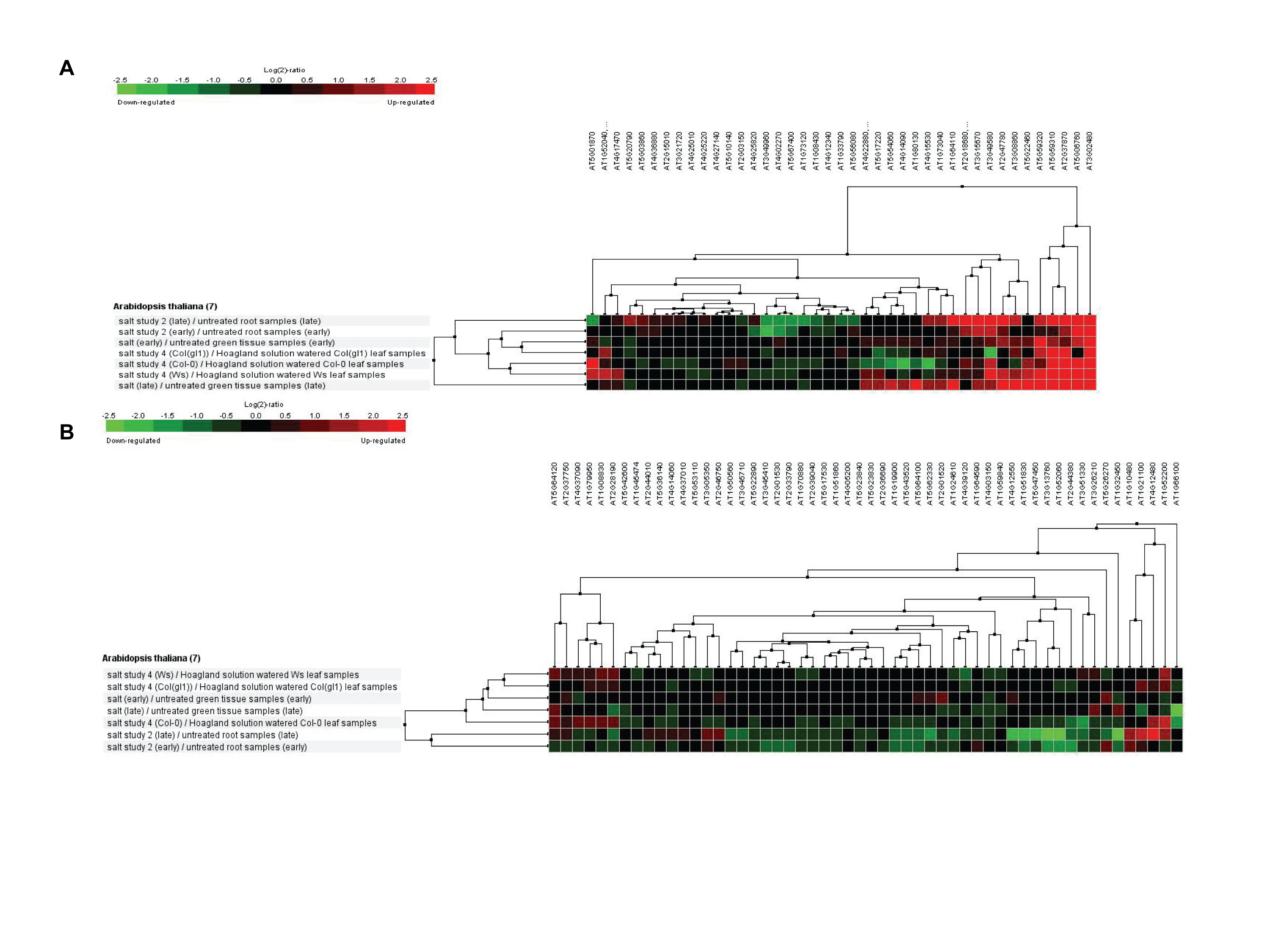

Supplement: Figure S5 — Hierarchical clustering analysis of genes in wild-type plants in response to salt stress treatments using publicly available gene expression data; these genes showed increased (A) and reduced (B) expression patterns in rsa1-1 under control conditions in our microarray analysis. Hierarchical clustering analysis was performed in Genevestigator with Hierarchical Clustering Tool (https://www.genevestigator.com/gv/user/gvLogin.jsp) [64]. Scale bars at the top indicate the relative expression level (green, repression; red, induction) of a gene compared to the non-stressed condition in wild-type plants. (TIF) [file pgen.1003755.s005.tif]

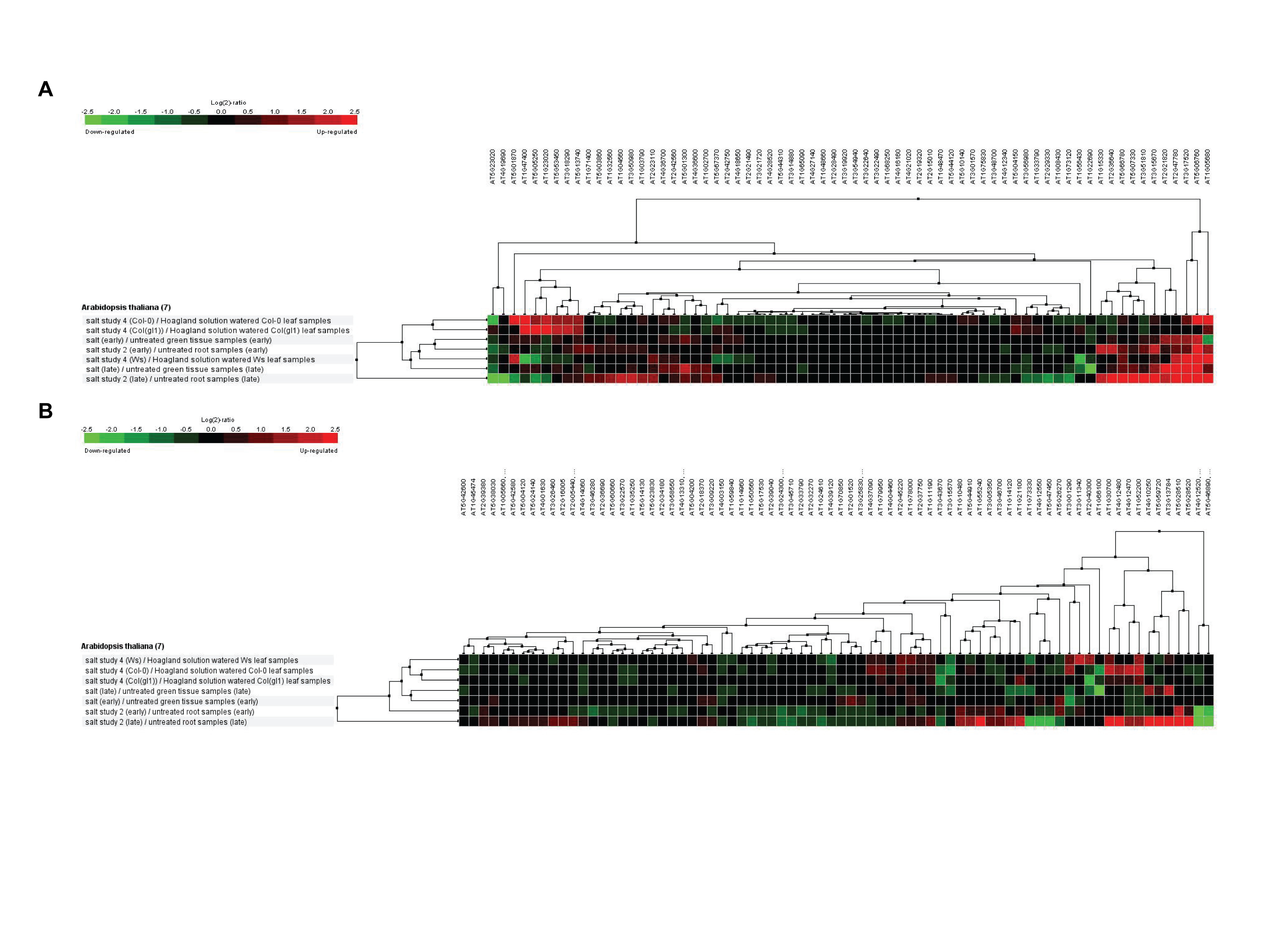

Supplement: Figure S6 — Hierarchical clustering analysis of genes in wild type plants in response to salt stress treatments using publicly available gene expression data; these genes showed increased (A) and reduced (B) expression patterns in rsa1-1 under salt stress in our microarray analysis. Hierarchical clustering analysis was performed in Genevestigator with Hierarchical Clustering Tool (https://www.genevestigator.com/gv/user/gvLogin.jsp) [64]. Scale bars at the top indicate the relative expression level (green, repression; red, induction) of a gene compared to the non-stressed condition in wild-type plants. (TIF) [file pgen.1003755.s006.tif]

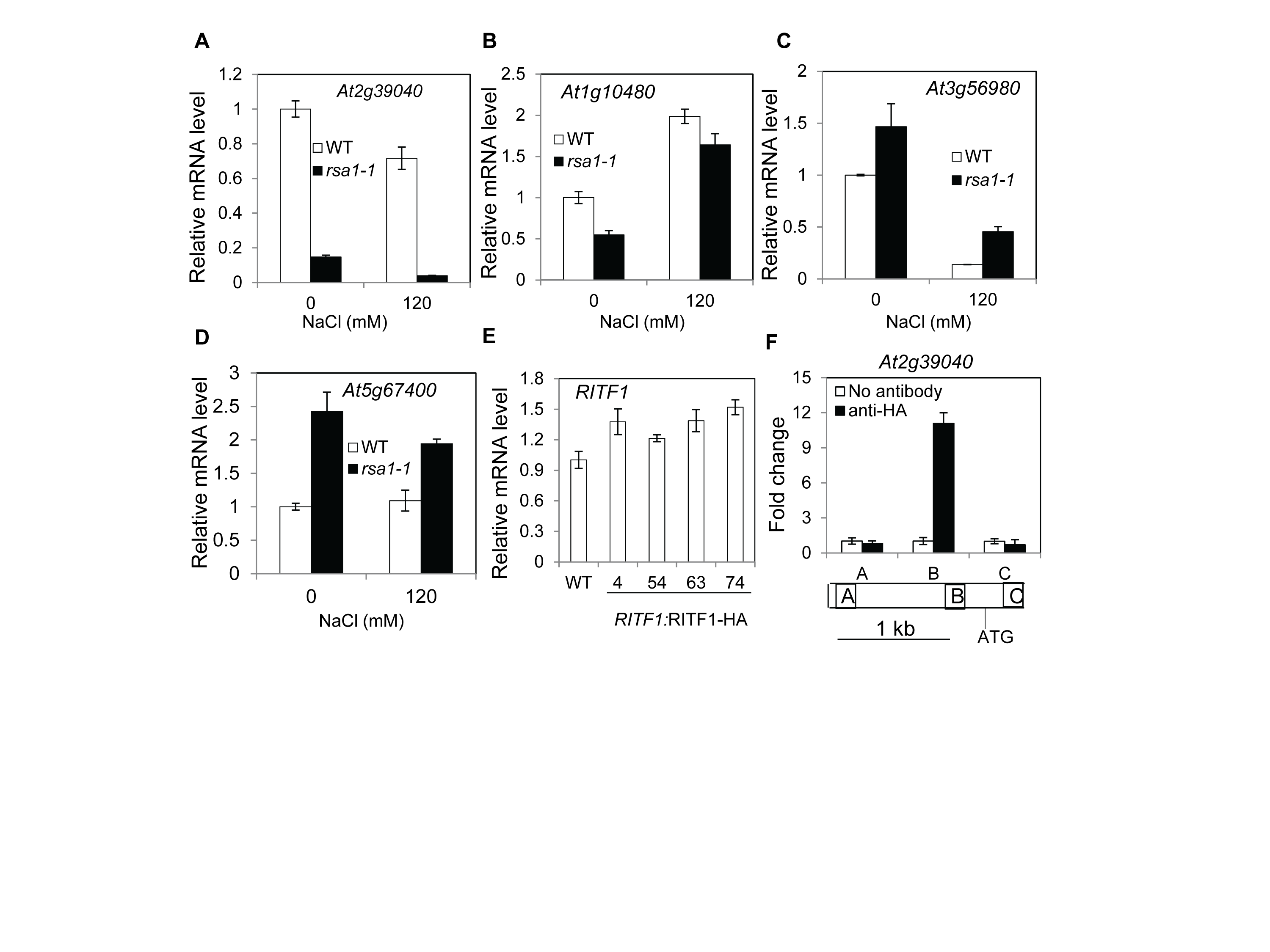

Supplement: Figure S7 — Validation of microarray results by qRT-PCR analysis and ChIP-qPCR analysis of the At2g39040 gene. (A)–(D) Validation of microarray results by qRT-PCR. Six-d-old wild-type and rsa1-1 seedlings grown on MS medium were transferred to MS medium containing 0 or 120 mM NaCl and allowed to grow for an additional 24 h. WT, wild type. At2g39040, At1g10480, At3g56980, and At5g67400 encode peroxidase, zinc finger protein 5, basic helix-loop-helix (bHLH) DNA-binding superfamily protein, and root hair specific 19 with putative peroxidase activity, respectively. (E) RITF1 expression in wild-type and transgenic plants expressing RITF1:RITF1-HA as determined by qRT-PCR analysis. Total RNA was isolated from 14-d-old seedlings grown on MS medium. (F) ChIP-qPCR analysis of three areas of the At2g39040 gene. Regions of amplification: A (containing two copies of core cis element CATATG at two different sites) = −833 to −588; B (containing two core cis elements CAATTG and CAAGTG) = −143 to +6; C (containing no core cis element; serving as negative control) = +171 to +269 base pairs relative to the translation start site. Error bars indicate the standard deviation (n = 4). The experiments in Figure S7 were performed at least three times with similar results, and data from one representative experiment are presented. (TIF) [file pgen.1003755.s007.tif]

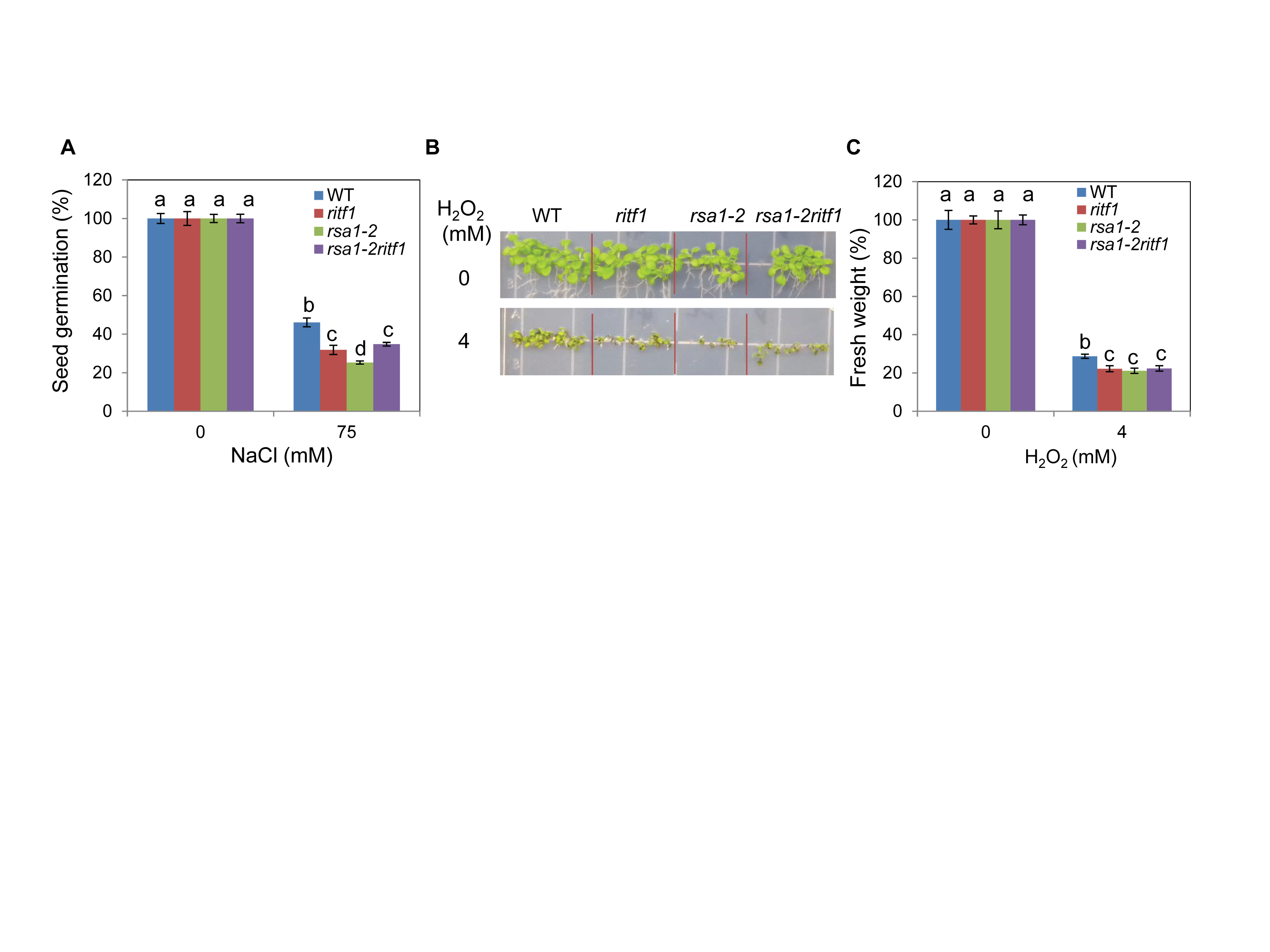

Supplement: Figure S8 — Responses of wild-type, ritf1, rsa1-2, and rsa1-2ritf1 seedlings to salt stress and oxidative stress. (A) Seed germination of wild type, ritf1, rsa1-2, and rsa1-2ritf1 double mutant under 75 mM NaCl treatment. There were 80–150 seeds per genotype per biological replicate. Seeds in which the radical had emerged through the seed coat were considered germinated. (B) Growth responses of wild-type, ritf1, rsa1-2, and rsa1-2ritf1 seedlings to H2O2. (C) Fresh weight of wild-type, ritf1, rsa1-2, and rsa1-2ritf1 seedlings under H2O2 treatment. In (B) and (C), seeds were sown directly on MS medium supplemented with various levels of H2O2 and allowed to grown for an additional 10 d. WT, wild type. One-way ANOVA (Tukey-Kramer test) was performed, and statistically significant differences are indicated by different lowercase letters (p<0.01). Error bars represent the standard deviation (n = 30–40). The experiments in Figure S8 were performed at least three times with similar results, and data from one representative experiment are presented. (TIF) [file pgen.1003755.s008.tif]

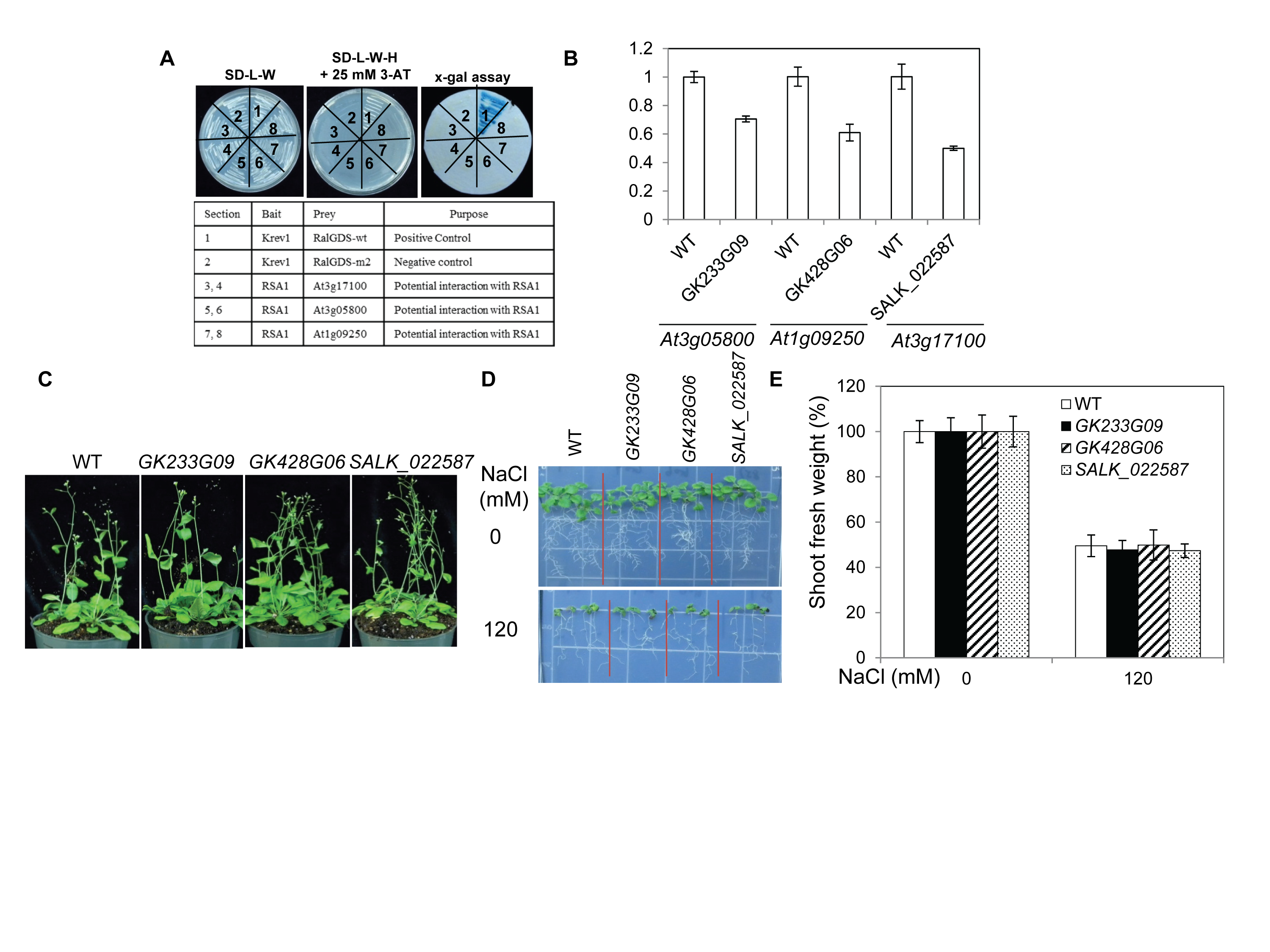

Supplement: Figure S9 — Yeast two-hybrid analysis of RSA1 and RITF1 homologs, and growth responses of the T-DNA mutant plants of RITF1 homologs under salt stress. (A) RSA1 does not interact with three close homologs of RITF1 as determined by yeast two-hybrid analysis. Yeast strain MaV203 co-transformed with different combinations of bait and prey was subjected to x-gal assay. Yeast cells grown on SD medium-L-W or SD medium-L-W-H+3-AT are shown. 3-AT, 3-amino-1,2,4-triazole. L, W, H, symbols for amino acids leucine, tryptophan, and histidine, respectively. SD, yeast minimal media. (B) Expression of At3g05800, At1g09250, or At3g17100 in the corresponding T-DNA mutant plants. GK233G09, GK428G06, and SALK_022587 are the T-DNA mutants of At3g05800, At1g09250, and At3g17100, respectively. qRT-PCR analysis was carried out with total RNA isolated from 14-d-old seedlings grown on MS medium. (C) Morphology of T-DNA mutant plants of RITF1 homologs. (D) Growth responses of T-DNA mutants of RITF1 homologs to salt stress. Five-d-old seedlings grown on MS medium were transferred to MS medium containing 0 or 120 mM NaCl and allowed to grow for an additional 7 d. (E) Shoot fresh weight of plants shown in (D). WT, wild type. Error bars represent the standard deviation (n = 4 in [B] and 40 in [E]). The experiments in Figure S9 were performed at least three times with similar results, and data from one representative experiment are presented. (TIF) [file pgen.1003755.s009.tif]

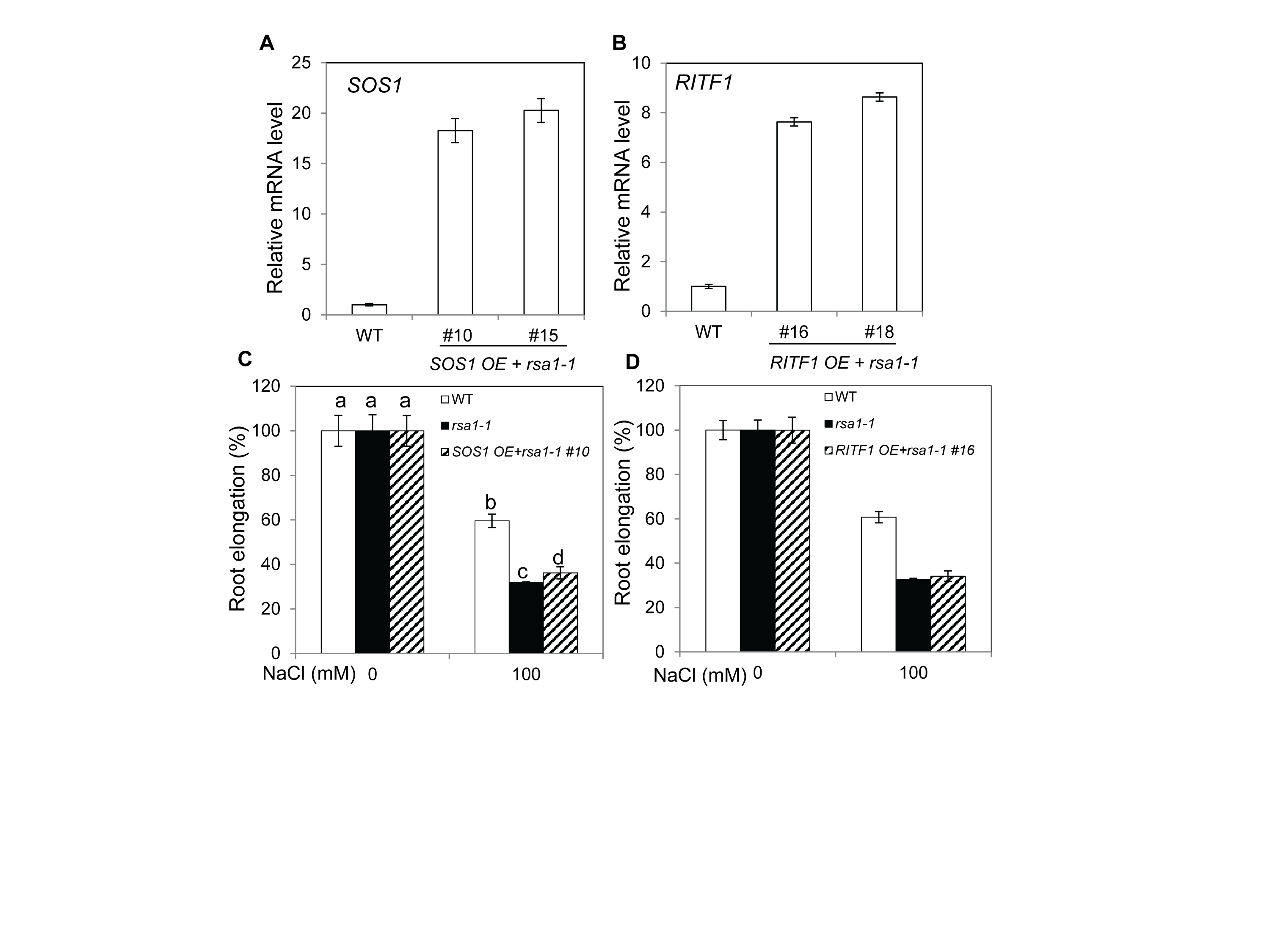

Supplement: Figure S10 — Phenotypes of rsa1-1 transgenic plants expressing p35S:SOS1 or p35S:RITF1. Five-d-old seedlings grown on MS medium were transferred to MS medium supplemented with various levels of NaCl and allowed to grow for an additional 10 d (for [C] and [D]). (A) SOS1 expression in wild-type and rsa1-1 plants expressing p35S:SOS1. (B) RITF1 expression in wild-type and rsa1-1 plants expressing p35S:RITF1. qRT-PCR analysis in (A) and (B) was performed with total RNA isolated from 14-d-old seedlings grown on MS medium. (C) Root elongation of wild-type, rsa1-1, and one representative line of rsa1-1 plants expressing p35S:SOS1 in response to 0 or 100 mM NaCl. (D) Root elongation of wild-type, rsa1-1, and one representative line of rsa1-1 plants expressing p35S:RITF1 in response to 0 or 100 mM NaCl. One-way ANOVA (Tukey-Kramer test) was performed, and statistically significant differences are indicated by different lowercase letters (p<0.01). Error bars represent the standard deviation (n = 4 in [A] and [B], 30–40 in [C] and [D]). The experiments in Figure S10 were performed at least three times with similar results, and data from one representative experiment are presented. (TIF) [file pgen.1003755.s010.tif]

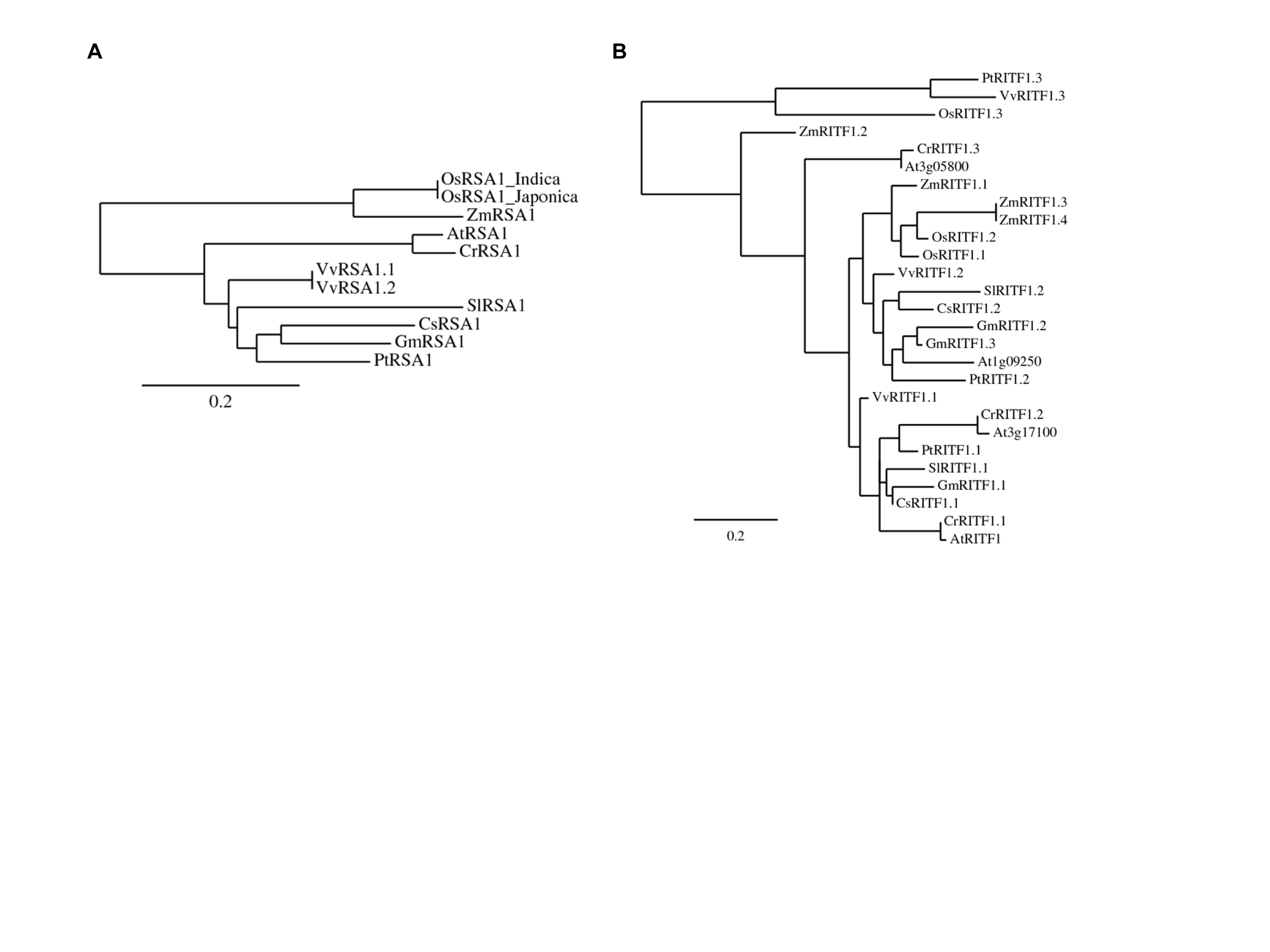

Supplement: Figure S11 — Comparison of AtRSA1 and AtRITF1 with their close homologs in other plant species. The phylogenetic tree was generated with the Phylogeny.fr platform (http://www.phylogeny.fr/version2_cgi/advanced.cgi) as described [65]. Scale bar indicates branch length. (A) Phylogenetic tree of AtRSA1 and its close homologs. The protein identities are as follow: AtRSA1 (Arabidopsis thaliana, NP_178414), ZmRSA1 (Zea mays, AFW76494), OsRSA1_Indica (Oryza sativa Indica Group, EEC80815), OsRSA1_Japonica (Oryza sativa Japonica Group, EEE65891), CrRSA1 (Capsella rubella, EOA23219), PtRSA1(Populus trichocarpa, XP_002316125), VvRSA1.1 (Vitis vinifera, CBI31934), VvRSA1.2 (Vitis vinifera, XP_002268851), SlRSA1 (Solanum lycopersicum, XP_004236885), GmRSA1(Glycine max, XP_003520085), and CsRSA1 (Cucumis sativus, XP_004143774). (B) Phylogenetic tree of AtRITF1 and its close homologs. The protein identities are as follow: AtRITF1 (Arabidopsis thaliana, NP_566287), At3g05800 (Arabidopsis thaliana, NP_566260), At1g09250 (Arabidopsis thaliana, NP_563839), At3g17100 (Arabidopsis thaliana, NP_566567), CsRITF1.1 (Cucumis sativus, XP_004148798), CsRITF1.2 (Cucumis sativus, XP_004135755), GmRITF1.1 (Glycine max, XP_003550916), GmRITF1.2 (Glycine max, XP_003520791), GmRITF1.3 (Glycine max, XP_003536039), SlRITF1.1 (Solanum lycopersicum, XP_004229017), SlRITF1.2 (Solanum lycopersicum, XP_004230318), VvRITF1.1 (Vitis vinifera, XP_002279307), VvRITF1.2 (Vitis vinifera, XP_002281846), VvRITF1.3 (Vitis vinifera, XP_002270621), PtRITF1.1 (Populus trichocarpa, XP_002312320), PtRITF1.2 (Populus trichocarpa, XP_002330080), PtRITF1.3 (Populus trichocarpa, XP_002325095),), CrRITF1.1 (Capsella rubella, EOA31396), CrRITF1.2 (Capsella rubella, EOA31360), CrRITF1.3 (Capsella rubella, EOA29635), OsRITF1.1 (Oryza sativa Indica, EAY89737), OsRITF1.2 (Oryza sativa Japonica, BAC21355), OsRITF1.3 (Oryza sativa Japonica, NP_001172473), ZmRITF1.1 (Zea mays, NP_001142628), ZmRITF1.2 (Zea mays, AFW80198), ZmRITF1.3 (Zea mays, [file pgen.1003755.s011.tif]
