# Supplementary material for: A Nuclear Calcium-Sensing Pathway Is Critical for Gene Regulation and Salt Stress Tolerance in Arabidopsis
Source: PLoS Genet. 2013 Aug 29;9(8):e1003755. doi: 10.1371/journal.pgen.1003755 (PMC3757082; doi:10.1371/journal.pgen.1003755)
Supplement: Table S1 — Genetic analysis of the rsa1-1 mutant (wild type [female]×rsa1-1 [male] cross). (PDF) [file pgen.1003755.s012.pdf]

Table S1. Genetic analysis of the *rsa1-1* mutant (wild type [female] x *rsa1-1* [male] cross).

Generation	Total plants tested	Wild type	*rsa1-1*	χ^2	P value
F_1	42	42	0		
F_2	478	366	112	0.63	$0.30 < P < 0.50$
