# Supplementary material for: A Nuclear Calcium-Sensing Pathway Is Critical for Gene Regulation and Salt Stress Tolerance in Arabidopsis
Source: PLoS Genet. 2013 Aug 29;9(8):e1003755. doi: 10.1371/journal.pgen.1003755 (PMC3757082; doi:10.1371/journal.pgen.1003755)
Supplement: Table S2 — Differentially expressed genes in rsa1-1 under the control condition as determined by microarray analysis. (A) Genes with increased expression in rsa1-1 without stress as determined by microarray analysis. (B) Genes with reduced expression in rsa1-1 without stress as determined by microarray analysis. (PDF) [file pgen.1003755.s013.pdf]

Table S2. Differentially expressed genes in *rsa1-1* under control condition as determined by microarray analysis.
Table S2A. Genes with increased expression in *rsa1-1* without stress as determined by microarray analysis.

AGI ID	Fold Change	P Value	Gene Description	Go Term (Biological Process)
AT1G64110	2.42	0.000017	P-loop containing nucleoside triphosphate hydrolases superfamily protein	developmental processes
AT2G03150	2.37	0.000024	ATP/GTP-binding protein family	developmental processes
AT3G15670	2.42	0.001547	Late embryogenesis abundant protein (LEA) family protein	developmental processes
AT1G08430	2.19	0.000142	aluminum-activated malate transporter 1	other biological processes
AT1G33790	3.06	0.000025	jacalin lectin family protein	other biological processes
AT1G73040	2.11	0.002153	Mannose-binding lectin superfamily protein	other biological processes
AT4G12340	3.36	0.000001	copper ion binding	other biological processes
AT2G47780	5.32	0.000002	Rubber elongation factor protein (REF)	other biological processes
AT3G08860	2.88	0.000005	PYRIMIDINE 4	other cellular processes
AT3G49580	2.51	0.004612	response to low sulfur 1	other cellular processes
AT4G25820	2.19	0.000805	xyloglucan endotransglucosylase/hydrolase 14	other cellular processes
AT5G22460	2.14	0.000098	alpha/beta-Hydrolases superfamily protein	other cellular processes
AT4G02270	2.18	0.000381	root hair specific 13	other enzyme activity
AT3G21720	3.16	0.000001	isocitrate lyase	other metabolic processes
AT4G14090	2.25	0.002155	UDP-Glycosyltransferase superfamily protein	other metabolic processes
AT4G15530	2.08	0.000085	pyruvate orthophosphate dikinase	other metabolic processes
AT4G22870	2.83	0.001225	2-oxoglutarate (2OG) and Fe(II)-dependent oxygenase superfamily protein	other metabolic processes
AT5G03860	3.16	0.000011	malate synthase	other metabolic processes
AT5G17220	2.85	0.002604	glutathione S-transferase phi 12	other metabolic processes
AT5G56080	2.14	0.000825	nicotianamine synthase 2	other metabolic processes
AT4G17470	2.33	0.000098	alpha/beta-Hydrolases superfamily protein	protein metabolism
AT5G54060	2.28	0.001335	UDP-glucose:flavonoid 3-o-glucosyltransferase	protein metabolism
AT3G02480	2.47	0.000049	Late embryogenesis abundant protein (LEA) family protein	response to abiotic or biotic stimulus
AT4G36880	2.69	0.000532	cysteine proteinase1	response to abiotic or biotic stimulus
AT5G06760	2.01	0.002845	Late Embryogenesis Abundant 4-5	response to abiotic or biotic stimulus
AT5G10140	4.39	0.000000	K-box region and MADS-box transcription factor family protein	response to abiotic or biotic stimulus
AT5G59310	6.33	0.000001	lipid transfer protein 4	response to abiotic or biotic stimulus
AT5G59320	4.13	0.000001	lipid transfer protein 3	response to abiotic or biotic stimulus
AT1G52040	3.74	0.002647	myrosinase-binding protein 1	response to stress
AT1G73120	2.46	0.000078	unknown protein	response to stress

AT1G80130	2.05	0.000046	Tetratricopeptide repeat (TPR)-like superfamily protein	response to stress
AT2G15010	3.29	0.001390	Plant thionin	response to stress
AT3G49960	2.24	0.002709	Peroxidase superfamily protein	response to stress
AT5G67400	2.33	0.002752	root hair specific 19	response to stress
AT2G37870	3.24	0.000000	seed storage 2S albumin superfamily protein	transport
AT4G25010	2.15	0.000238	Nodulin MtN3 family protein	transport
AT4G25220	2.35	0.000135	root hair specific 15	transport
AT4G27140	3.69	0.000004	seed storage albumin 1	transport
AT5G01870	3.13	0.000004	seed storage 2S albumin superfamily protein	transport
AT2G18680	2.47	0.000173	unknown protein	unknown biological processes
AT5G20790	2.06	0.000056	unknown protein	unknown biological processes

Table S2B. Genes with reduced expression in *rsa1-1* without stress as determined by microarray analysis.

AGI ID	Fold Change	P Value	Gene Description	Go Term (Biological Process)
AT1G59840	4.77	0.000001	cofactor assembly of complex C	cell organization and biogenesis
AT4G12550	4.56	0.000016	Auxin-Induced in Root cultures 1	developmental processes
AT1G45474	2.16	0.000008	photosystem I light harvesting complex gene 5	electron transport or energy pathways
AT2G33790	4.33	0.000000	arabinogalactan protein 30	other biological processes
AT5G23830	2.00	0.000401	MD-2-related lipid recognition domain-containing protein	other cellular processes
AT5G23840	2.66	0.000349	MD-2-related lipid recognition domain-containing protein	other cellular processes
AT5G53110	2.03	0.000532	RING/U-box superfamily protein	other cellular processes
AT1G21100	5.28	0.000004	O-methyltransferase family protein	other metabolic processes
AT1G50560	2.06	0.000263	cytochrome P450 family protein (CYP705A25)	other metabolic processes
AT1G64590	2.32	0.000976	NAD(P)-binding Rossmann-fold superfamily protein	other metabolic processes
AT2G44380	3.14	0.000001	Cysteine/Histidine-rich C1 domain family protein	other metabolic processes
AT2G46750	2.88	0.000068	D-arabinono-1,4-lactone oxidase family protein	other metabolic processes
AT3G13760	2.30	0.002161	Cysteine/Histidine-rich C1 domain family protein	other metabolic processes
AT3G26210	2.46	0.000187	cytochrome P450, family 71, subfamily B, polypeptide 23	other metabolic processes
AT4G39120	3.61	0.000008	myo-inositol monophosphatase like 2	other metabolic processes
AT5G17530	2.73	0.000001	phosphoglucosamine mutase family protein	other metabolic processes
AT5G36140	2.39	0.000381	cytochrome P450, family 716, subfamily A, polypeptide 2	other metabolic processes
AT5G42600	3.44	0.000177	marneral synthase	other metabolic processes

AT5G43520	2.22	0.000143	Cysteine/Histidine-rich C1 domain family protein	other metabolic processes
AT1G19900	2.68	0.000010	glyoxal oxidase-related protein	other metabolic processes
AT1G24610	2.27	0.000001	Rubisco methyltransferase family protein	other metabolic processes
AT1G52060	2.08	0.000143	Mannose-binding lectin superfamily protein	other metabolic processes
AT2G36690	2.0315	0.00139035	2-oxoglutarate (2OG) and Fe(II)-dependent oxygenase superfamily protein	other metabolic processes
AT5G62330	2.48	0.000970	Plant invertase/pectin methylesterase inhibitor superfamily protein	other metabolic processes
AT1G51860	2.03	0.000099	Leucine-rich repeat protein kinase family protein	protein metabolism
AT3G05350	2.22	0.000004	Metallopeptidase M24 family protein	protein metabolism
AT3G51330	2.03	0.001389	Eukaryotic aspartyl protease family protein	protein metabolism
AT4G05200	2.44	0.000087	cysteine-rich RLK (RECEPTOR-like protein kinase) 25	protein metabolism
AT1G08830	2.15	0.004902	copper/zinc superoxide dismutase 1	response to abiotic or biotic stimulus
AT1G70880	2.03	0.000262	Polyketide cyclase/dehydrase and lipid transport superfamily protein	response to abiotic or biotic stimulus
AT2G01520	2.96	0.001662	MLP-like protein 328	response to abiotic or biotic stimulus
AT2G01530	2.57	0.000010	MLP-like protein 329	response to abiotic or biotic stimulus
AT2G28190	2.51	0.000397	copper/zinc superoxide dismutase 2	response to abiotic or biotic stimulus
AT3G45410	2.16	0.000537	Concanavalin A-like lectin protein kinase family protein	response to abiotic or biotic stimulus
AT4G12480	2.06	0.000433	seed storage 2S albumin superfamily protein	response to abiotic or biotic stimulus
AT4G14060	2.75	0.000171	Polyketide cyclase/dehydrase and lipid transport superfamily protein	response to abiotic or biotic stimulus
AT5G47450	2.59	0.000041	tonoplast intrinsic protein 2;3	response to abiotic or biotic stimulus
AT5G64120	2.89	0.000011	Peroxidase superfamily protein	response to abiotic or biotic stimulus
AT1G52200	2.81	0.000005	PLAC8 family protein	response to stress
AT1G66100	3.66	0.000062	Plant thionin	response to stress
AT2G39040	2.03	0.000020	Peroxidase superfamily protein	response to stress
AT4G37010	2.06	0.003164	centrin 2	response to stress
AT5G64100	2.12	0.000430	Peroxidase superfamily protein	response to stress
AT1G10480	1.5936	0.001672	zinc finger protein 5	signal transduction
AT1G51830	2.07	0.000201	Leucine-rich repeat protein kinase family protein	signal transduction
AT1G79950	2.73	0.000130	RAD3-like DNA-binding helicase protein	transcription,DNA-dependent
AT5G22890	2.17	0.000245	C2H2 and C2HC zinc fingers superfamily protein	transcription,DNA-dependent
AT1G32450	2.47	0.000004	nitrate transporter 1.5	transport
AT3G45710	2.35	0.000098	Major facilitator superfamily protein	transport
AT2G37750	2.21	0.000025	unknown protein	unknown biological processes
AT2G44010	2.34	0.000031	unknown protein	unknown biological processes
AT4G03150	2.79	0.000170	unknown protein	unknown biological processes
AT4G37090	2.04	0.001050	unknown protein	unknown biological processes
AT5G26270	2.87	0.000020	unknown protein	unknown biological processes
