# Supplementary material for: A Nuclear Calcium-Sensing Pathway Is Critical for Gene Regulation and Salt Stress Tolerance in Arabidopsis
Source: PLoS Genet. 2013 Aug 29;9(8):e1003755. doi: 10.1371/journal.pgen.1003755 (PMC3757082; doi:10.1371/journal.pgen.1003755)
Supplement: Table S3 — Differentially expressed genes in rsa1-1 under salt stress as determined by microarray analysis. (A) Genes with increased expression in rsa1-1 under salt stress as determined by microarray analysis. (B) Genes with reduced expression in rsa1-1 under salt stress as determined by microarray analysis. (PDF) [file pgen.1003755.s014.pdf]

Table S3. Differentially expressed genes in *rsa1-1* under salt stress as determined by microarray analysis.

Table S3A. Genes with increased expression in *rsa1-1* under salt stress as determined by microarray analysis.

AGI ID	Fold Change	P Value	Gene Description	Go Term (Biological Process)
AT1G15330	4.14	0.000002	Cystathionine beta-synthase (CBS) protein	developmental processes
AT2G36640	2.29	0.008510	embryonic cell protein 63	developmental processes
AT2G42560	2.04	0.003709	LEA domain-containing protein	developmental processes
AT3G15670	4.20	0.000018	Late embryogenesis abundant protein (LEA) family protein	developmental processes
AT3G17520	2.09	0.000251	Late embryogenesis abundant protein (LEA) family protein	developmental processes
AT3G22490	2.15	0.000591	Seed maturation protein	developmental processes
AT3G51810	3.42	0.000012	Stress induced protein	developmental processes
AT4G21020	2.43	0.000719	Late embryogenesis abundant protein (LEA) family protein	developmental processes
AT4G28520	2.18	0.000998	cruciferin 3	developmental processes
AT4G36600	2.84	0.002876	Late embryogenesis abundant (LEA) protein	developmental processes
AT5G44120	2.07	0.0004387	RmlC-like cupins superfamily protein	developmental processes
AT5G44310	2.63	0.0013813	Late embryogenesis abundant protein (LEA) family protein	developmental processes
AT1G08430	2.44	0.000033	aluminum-activated malate transporter 1	other biological processes
AT1G22690	2.12	0.000031	Gibberellin-regulated family protein	other biological processes
AT1G48660	2.58	0.000017	Auxin-responsive GH3 family protein	other biological processes
AT3G01570	2.68	0.000005	Oleosin family protein	other biological processes
AT2G28490	2.05	0.000800	RmlC-like cupins superfamily protein	other biological processes
AT2G42750	2.02	0.000537	DNAJ heat shock N-terminal domain-containing protein	other cellular processes
AT3G18290	2.26	0.000017	zinc finger protein-related	other cellular processes
AT1G04560	2.03	0.002847	AWPM-19-like family protein	other cellular processes
AT1G33790	2.95	0.000024	jacalin lectin family protein	other cellular processes
AT1G47400	5.10	0.000101	unknown protein	other cellular processes
AT2G47780	5.11	0.000002	Rubber elongation factor protein (REF)	other cellular processes
AT3G22640	3.06	0.007130	cupin family protein	other cellular processes
AT4G36700	2.25	0.000202	RmlC-like cupins superfamily protein	other cellular processes
AT4G12340	3.72	0.000000	copper ion binding	other cellular processes
AT1G23020	2.89	0.000007	ferric reduction oxidase 3	other metabolic processes
AT1G48470	2.19	0.000870	glutamine synthetase 1;5	other metabolic processes
AT1G56430	2.03	0.007985	nicotianamine synthase 4	other metabolic processes
AT2G29330	2.68	0.000413	tropinone reductase	other metabolic processes
AT3G21720	3.16	0.000001	isocitrate lyase	other metabolic processes
AT3G48700	2.20	0.000168	carboxyesterase 13	other metabolic processes

AT5G03860	4.97	0.000000	malate synthase	other metabolic processes
AT5G23020	2.04	0.0002730	2-isopropylmalate synthase 2	other metabolic processes
AT5G01300	2.02	0.000398	PEBP (phosphatidylethanolamine-binding protein) family protein	other metabolic processes
AT3G54940	2.05	0.001171	Papain family cysteine protease	protein metabolism
AT5G53450	2.94	0.0000218	OBP3-responsive gene 1	protein metabolism
AT1G05680	2.26	0.000054	Uridine diphosphate glycosyltransferase 74E2	response to abiotic or biotic stimulus
AT1G32560	2.17	0.002025	Late embryogenesis abundant protein 4-1	response to abiotic or biotic stimulus
AT2G21490	3.73	0.000096	dehydrin LEA	response to abiotic or biotic stimulus
AT3G14880	2.08	0.000041	transcription factor-related	response to abiotic or biotic stimulus
AT3G50980	2.19	0.000054	dehydrin xero 1	response to abiotic or biotic stimulus
AT4G19690	6.40	0.000004	iron-regulated transporter 1	response to abiotic or biotic stimulus
AT5G06760	2.10	0.001487	Late Embryogenesis Abundant 4-5	response to abiotic or biotic stimulus
AT5G10140	4.84	0.0000000	K-box region and MADS-box transcription factor family protein	response to abiotic or biotic stimulus
AT5G13740	2.05	0.0000623	zinc induced facilitator 1	response to abiotic or biotic stimulus
AT1G71400	2.37	0.000001	receptor like protein 12	response to stress
AT1G73120	2.11	0.000268	unknown protein	response to stress
AT1G75830	2.14	0.008293	low-molecular-weight cysteine-rich 67	response to stress
AT2G15010	4.78	0.000101	Plant thionin	response to stress
AT2G23110	2.44	0.000304	Late embryogenesis abundant protein, group 6	response to stress
AT3G18290	2.26	0.000017	zinc finger protein-related	response to stress
AT1G03790	2.01	0.000582	Zinc finger C-x8-C-x5-C-x3-H type family protein	transcription,DNA-dependent
AT3G56980	3.71	0.000019	basic helix-loop-helix (bHLH) DNA-binding superfamily protein	transcription,DNA-dependent
AT5G04150	5.57	0.000009	basic helix-loop-helix (bHLH) DNA-binding superfamily protein	transcription,DNA-dependent
AT4G18650	2.30	0.000496	transcription factor-related	transcription,DNA-dependent
AT4G16160	2.34	0.000041	Mitochondrial import inner membrane translocase subunit	transport
AT4G27140	12.48	0.000000	seed storage albumin 1	transport
AT5G01870	2.34	0.000041	lipid-transfer protein	transport
AT1G02700	2.24	0.000017	unknown protein	unknown biological processes
AT1G65090	3.28	0.000012	unknown protein	unknown biological processes
AT1G68250	2.75	0.000118	unknown protein	unknown biological processes
AT2G19320	2.19	0.000552	unknown protein	unknown biological processes
AT2G21820	2.19	0.002025	unknown protein	unknown biological processes
AT3G19920	2.20	0.000512	unknown protein	unknown biological processes
AT5G05250	2.45	0.000357	unknown protein	unknown biological processes
AT5G07330	2.53	0.0001317	unknown protein	unknown biological processes
AT5G66780	4.49	0.0000069	unknown protein	unknown biological processes
AT5G67370	2.21	0.0000664	unknown protein	unknown biological processes

Table S3B. Genes with reduced expression in *rsa1-1* under salt stress as determined by microarray analysis.

AGI ID	Fold Change	P Value	Gene Description	Go Term (Biological Process)
AT1G59840	5.34	0.000000	cofactor assembly of complex C	cell organization and biogenesis
AT2G45220	2.91	0.000007	Plant invertase/pectin methylesterase inhibitor superfamily	cell organization and biogenesis
AT4G01630	2.07	0.000069	expansin A17	cell organization and biogenesis
AT1G11190	2.58	0.000054	bifunctional nuclease i	DNA or RNA metabolism
AT1G45474	3.39	0.000000	photosystem I light harvesting complex gene 5	electron transport or energy pathways
AT2G05510	2.10	0.000130	Glycine-rich protein	other biological processes
AT2G16005	2.55	0.004500	MD-2-related lipid recognition domain-containing protein	other biological processes
AT2G33790	3.95	0.000000	arabinogalactan protein 30	other biological processes
AT5G23830	2.74	0.000012	MD-2-related lipid recognition domain-containing protein	other biological processes
AT1G21100	2.48	0.000632	O-methyltransferase family protein	other cellular processes
AT3G09220	2.16	0.000035	laccase 7	other cellular processes
AT1G05660	2.87	0.000066	Pectin lyase-like superfamily protein	other metabolic processes
AT1G14120	2.37	0.002630	2-oxoglutarate (2OG) and Fe(II)-dependent oxygenase superfamily protein	other metabolic processes
AT1G30700	2.49	0.000049	FAD-binding Berberine family protein	other metabolic processes
AT1G50560	2.16	0.000109	cytochrome P450, family 705, subfamily A, polypeptide 25	other metabolic processes
AT2G36690	2.08	0.000189	2-oxoglutarate (2OG) and Fe(II)-dependent oxygenase superfamily protein	other metabolic processes
AT3G13784	2.09	0.001985	cell wall invertase 5	other metabolic processes
AT3G25830	2.71	0.001655	terpene synthase-like sequence-1,8-cineole	other metabolic processes
AT3G43670	2.60	0.000133	Copper amine oxidase family protein	other metabolic processes
AT3G46700	2.13	0.000591	UDP-Glycosyltransferase superfamily protein	other metabolic processes
AT4G13310	2.82	0.000005	cytochrome P450, family 71, subfamily A, polypeptide 20	other metabolic processes
AT4G39120	5.96	0.000000	myo-inositol monophosphatase like 2	other metabolic processes
AT5G04120	3.04	0.000206	Phosphoglycerate mutase family protein	other metabolic processes
AT5G17530	3.68	0.000000	phosphoglucosamine mutase family protein	other metabolic processes
AT5G24140	2.03	0.001785	squalene monooxygenase 2	other metabolic processes
AT5G28510	2.98	0.000000	beta glucosidase 24	other metabolic processes
AT5G42580	2.29	0.002919	cytochrome P450, family 705, subfamily A, polypeptide 12	other metabolic processes
AT5G42600	2.69	0.000857	marneral synthase	other metabolic processes
AT1G24610	2.63	0.000000	Rubisco methyltransferase family protein	other metabolic processes
AT1G35250	2.23	0.000041	Thioesterase superfamily protein	other metabolic processes
AT5G28520	3.87	0.000018	Mannose-binding lectin superfamily protein	other metabolic processes
AT3G05350	2.52	0.000001	Metallopeptidase M24 family protein	protein metabolism
AT3G46280	2.35	0.000048	protein kinase-related	protein metabolism

AT4G04460	2.16	0.000052	Saposin-like aspartyl protease family protein	protein metabolism
AT5G04200	2.00	0.000005	metacaspase 9	protein metabolism
AT1G14960	2.05	0.016870	Polyketide cyclase/dehydrase and lipid transport superfamily protein	response to abiotic or biotic stimulus
AT1G70850	2.02	0.000142	MLP-like protein 34	response to abiotic or biotic stimulus
AT1G73330	2.10	0.000241	drought-repressed 4	response to abiotic or biotic stimulus
AT2G01520	2.92	0.001365	MLP-like protein 328	response to abiotic or biotic stimulus
AT3G01290	2.01	0.000504	SPFH/Band 7/PHB domain-containing membrane-associated protein family	response to abiotic or biotic stimulus
AT3G11340	2.28	0.000010	UDP-Glycosyltransferase superfamily protein	response to abiotic or biotic stimulus
AT3G15570	2.79	0.000315	Phototropic-responsive NPH3 family protein	response to abiotic or biotic stimulus
AT3G26460	3.23	0.000071	Polyketide cyclase/dehydrase and lipid transport superfamily protein	response to abiotic or biotic stimulus
AT4G10250	2.06	0.000385	HSP20-like chaperones superfamily protein	response to abiotic or biotic stimulus
AT4G12470	2.01	0.009553	azelaic acid induced 1	response to abiotic or biotic stimulus
AT4G12480	2.20	0.000135	lipid-transfer protein	response to abiotic or biotic stimulus
AT4G14060	2.03	0.002251	Polyketide cyclase/dehydrase and lipid transport superfamily protein	response to abiotic or biotic stimulus
AT5G38030	2.31	0.000023	MATE efflux family protein	response to abiotic or biotic stimulus
AT5G47450	2.97	0.000009	tonoplast intrinsic protein 2;3	response to abiotic or biotic stimulus
AT5G59720	4.03	0.000001	heat shock protein 18.2	response to abiotic or biotic stimulus
AT1G52200	2.95	0.000003	PLAC8 family protein	response to stress
AT1G66100	3.11	0.000128	Plant thionin	response to stress
AT1G78000	2.00	0.000012	sulfate transporter 1;2	response to stress
AT2G40300	2.05	0.000034	ferritin 4	response to stress
AT5G14130	2.16	0.000017	Peroxidase superfamily protein	response to stress
AT5G44910	2.18	0.000681	Toll-Interleukin-Resistance (TIR) domain family protein	response to stress
AT2G39040	1.92	0.000027	Peroxidase superfamily protein	response to stress
AT1G10480	2.18	0.000017	zinc finger protein 5	signal transduction
AT2G34180	2.19	0.000229	CBL-interacting protein kinase 13	signal transduction
AT1G79950	2.68	0.000104	RAD3-like DNA-binding helicase protein	transcription,DNA-dependent
AT2G18370	2.13	0.000012	lipid-transfer protein	transport
AT2G32270	2.27	0.000023	zinc transporter 3 precursor	transport
AT2G39380	2.36	0.000007	exocyst subunit exo70 family protein H2	transport
AT3G22570	2.01	0.000026	lipid-transfer protein	transport
AT3G24300	2.41	0.000026	ammonium transporter 1;3	transport
AT3G45710	2.16	0.000168	Major facilitator superfamily protein	transport
AT3G58550	2.14	0.000067	lipid-transfer protein	transport
AT4G12510	2.08	0.000049	lipid-transfer protein	transport
AT4G12550	5.77	0.000004	Auxin-Induced in Root cultures 1	transport
AT5G46900	2.21	0.000140	lipid-transfer protein	transport
AT5G60660	2.31	0.000020	plasma membrane intrinsic protein 2;4	transport
AT1G55240	2.40	0.000049	unknown protein	unknown biological processes

AT2G37750	2.49	0.000005	unknown protein	unknown biological processes
AT4G03150	3.12	0.000048	unknown protein	unknown biological processes
AT4G37090	3.03	0.000018	unknown protein	unknown biological processes
AT5G26270	2.52	0.000047	unknown protein	unknown biological processes
