# Supplementary material for: A Nuclear Calcium-Sensing Pathway Is Critical for Gene Regulation and Salt Stress Tolerance in Arabidopsis
Source: PLoS Genet. 2013 Aug 29;9(8):e1003755. doi: 10.1371/journal.pgen.1003755 (PMC3757082; doi:10.1371/journal.pgen.1003755)
Supplement: Table S4 — Genes that are differentially expressed in rsa1-1 under both control and salt stress conditions as determined by microarray analysis. (A) Genes with increased expression in rsa1-1 under both control and salt stress conditions as determined by microarray analysis. (B) Genes with reduced expression in rsa1-1 under both control and salt stress conditions as determined by microarray analysis. (PDF) [file pgen.1003755.s015.pdf]

Table S4. **Genes that are differentially expressed in *rsa1-1* under both control and salt stress conditions as determined by microarray analysis.**

Table S4A. **Genes with increased expression in *rsa1-1* under both control and salt stress conditions as determined by microarray analysis.**

AGI ID	Fold Change (Control)	P Value (Control)	Fold Change (Salt Stress)	P Value (Salt Stress)	Gene Description	Go Term (Biological Process)
AT3G15670	2.42	0.0015472	4.20	0.0000183	Late embryogenesis abundant protein (LEA) family protein	developmental processes
AT1G08430	2.19	0.0001419	2.44	0.0000333	aluminum-activated malate transporter 1	other biological processes
AT1G33790	3.06	0.0000249	2.95	0.0000245	jacalin lectin family protein	other cellular processes
AT2G47780	5.32	0.0000023	5.11	0.0000025	Rubber elongation factor protein (REF)	other cellular processes
AT4G12340	3.36	0.0000007	3.72	0.0000002	copper ion binding	other cellular processes
AT3G21720	3.16	0.0000007	3.16	0.0000006	isocitrate lyase	other metabolic processes
AT5G03860	3.16	0.0000107	4.97	0.0000003	malate synthase	other metabolic processes
AT5G06760	2.01	0.0028449	2.10	0.0014866	Late Embryogenesis Abundant 4-5	response to abiotic or biotic stimulus
AT5G10140	4.39	0.0000000	4.84	0.0000000	K-box region and MADS-box transcription factor family protein	response to abiotic or biotic stimulus
AT1G73120	2.46	0.0000777	2.11	0.0002681	unknown protein	response to stress
AT2G15010	3.29	0.0013903	4.78	0.0001008	Plant thionin	response to stress
AT4G27140	3.69	0.0000037	12.48	0.0000000	seed storage albumin 1	transport
AT5G01870	3.13	0.0000037	2.34	0.0000413	lipid-transfer protein	transport

Table S4B. **Genes with reduced expression in *rsa1-1* under both control and salt stress conditions as determined by microarray analysis.**

AGI ID	Fold Change (Control)	P Value (Control)	Fold Change (Salt Stress)	P Value (Salt Stress)	Gene Description	Go Term (Biological Process)
AT1G59840	4.765	0.0000007	5.339	0.0000002	cofactor assembly of complex C	cell organization and biogenesis
AT1G45474	2.162	0.0000080	3.386	0.0000001	photosystem I light harvesting complex gene 5	electron transport or energy pathways
AT2G33790	4.333	0.0000001	3.947	0.0000001	arabinogalactan protein 30	other biological processes
AT5G23830	2.002	0.0004012	2.744	0.0000117	MD-2-related lipid recognition domain-containing protein	other biological processes
AT1G21100	5.277	0.0000037	2.482	0.0006319	O-methyltransferase family protein	other cellular processes
AT1G50560	2.062	0.0002629	2.158	0.0001091	cytochrome P450, family 705, subfamily A, polypeptide 25	other metabolic processes
AT4G39120	3.609	0.0000082	5.959	0.0000002	myo-inositol monophosphatase like 2	other metabolic processes
AT5G17530	2.725	0.0000009	3.681	0.0000001	phosphoglucosamine mutase family protein	other metabolic processes
AT5G42600	3.443	0.0001774	2.686	0.0008573	marneral synthase	other metabolic processes
AT1G24610	2.274	0.0000009	2.63	0.0000002	Rubisco methyltransferase family protein	other metabolic processes
AT2G36690	2.032	0.0013903	2.079	0.0001887	2-oxoglutarate (2OG) and Fe(II)-dependent oxygenase superfamily protein	other metabolic processes
AT3G05350	2.218	0.0000037	2.522	0.0000006	Metallopeptidase M24 family protein	protein metabolism

AT2G01520	2.958	0.0016625	2.923	0.0013653	MLP-like protein 328	response to abiotic or biotic stimulus
AT4G12480	2.055	0.0004326	2.201	0.0001352	lipid-transfer protein	response to abiotic or biotic stimulus
AT4G14060	2.746	0.0001713	2.035	0.0022514	Polyketide cyclase/dehydrase and lipid transport superfamily protein	response to abiotic or biotic stimulus
AT5G47450	2.585	0.0000412	2.972	0.0000089	tonoplast intrinsic protein 2;3	response to abiotic or biotic stimulus
AT1G52200	2.81	0.0000054	2.954	0.0000032	PLAC8 family protein	response to stress
AT1G66100	3.657	0.0000615	3.112	0.0001280	Plant thionin	response to stress
AT2G39040	2.032	0.0000201	1.924	0.0000270	Peroxidase superfamily protein	response to stress
AT1G10480	1.594	0.0016722	2.181	0.0000165	zinc finger protein 5	signal transduction
AT1G79950	2.734	0.0001302	2.676	0.0001039	RAD3-like DNA-binding helicase protein	transcription,DNA-dependent
AT4G12550	4.556	0.0000162	5.766	0.0000036	Auxin-Induced in Root cultures 1	transport
AT3G45710	2.352	0.0000984	2.156	0.0001684	Major facilitator superfamily protein	transport
AT2G37750	2.206	0.0000245	2.49	0.0000054	unknown protein	unknown biological processes
AT4G03150	2.792	0.0001695	3.121	0.0000477	unknown protein	unknown biological processes
AT4G37090	2.043	0.0010499	3.031	0.0000183	unknown protein	unknown biological processes
AT5G26270	2.868	0.0000201	2.517	0.0000474	unknown protein	unknown biological processes
