# Supplementary material for: A Nuclear Calcium-Sensing Pathway Is Critical for Gene Regulation and Salt Stress Tolerance in Arabidopsis
Source: PLoS Genet. 2013 Aug 29;9(8):e1003755. doi: 10.1371/journal.pgen.1003755 (PMC3757082; doi:10.1371/journal.pgen.1003755)
Supplement: Table S5 — Putative binding sites of bHLH transcription factors (TFs) in promoters of genes differentially expressed in rsa1-1 with or without salt stress as revealed by microarray analysis. (A) Putative bHLH TFs binding sites in promoters of genes with increased expression in rsa1-1 without stress. (B) Putative bHLH TFs binding sites in promoters of genes with reduced expression in rsa1-1 without stress. (C) Putative bHLH TFs binding sites in promoters of genes with increased expression in rsa1-1 under salt stress. (D) Putative bHLH TFs binding sites in promoters of genes with reduced expression in rsa1-1 under salt stress. (PDF) [file pgen.1003755.s016.pdf]

Table S5. Putative binding sites of bHLH transcription factors (TFs) in promoters of genes differentially expressed in *rsa1-1* with or without salt stress as revealed by microarray analysis.

Table S5A. Putative bHLH TFs binding sites in promoters of genes with increased expression in *rsa1-1* without etress.

Motif	Aboundance	Number of genes containing this motif	Total number of genes searched
CAAATG	92	35	41
CAACTG	37	23	41
CAAGTG	58	30	41
CAATTG	66	23	41
CACATG	62	29	41
CACCTG	20	15	41
CACGTG	60	18	41
CACTTG	58	30	41
CAGATG	39	26	41
CAGCTG	34	15	41
CAGGTG	20	15	41
CAGTTG	37	23	41
CATATG	88	23	41
CATCTG	39	26	41
CATGTG	62	29	41
CATTTG	92	35	41

Table S5B. Putative bHLH TFs binding sites in promoters of genes with reduced expression in *rsa1-1* without etress.

Motif	Aboundance	Number of genes containing this motif	Total number of genes searched
CAAATG	139	51	54
CAACTG	48	29	54
CAATTG	90	28	54
CACATG	84	40	54
CACCTG	29	21	54
CACGTG	36	16	54
CACTTG	62	34	54
CAGATG	62	34	54
CAGCTG	50	21	54
CAGGTG	29	21	54
CAGTTG	48	29	54
CAGTTG	48	29	54
CATATG	122	36	54
CATCTG	62	34	54
CATGTG	84	40	54
CATTTG	139	51	54

Table S5C. Putative bHLH TFs binding sites in promoters of genes with increased expression in *rsa1-1* under salt stress.

Motif	Aboundance	Number of genes containing this motif	Total number of genes searched
CAAATG	180	66	69
CAACTG	65	45	69
CAAGTG	113	55	69
CAATTG	112	39	69
CACATG	126	56	69
CACCTG	39	26	69
CACGTG	138	40	69
CACTTG	113	55	69
CAGATG	78	46	69
CAGCTG	64	24	69
CAGGTG	39	26	69
CAGTTG	65	45	69
CATATG	138	41	69
CATCTG	78	46	69
CATGTG	126	56	69
CATTTG	180	66	69

Table S5D. Putative bHLH TFs binding sites in promoters of genes with reduced expression in *rsa1-1* under salt stress.

Motif	Aboundance	Number of genes containing this motif	Total number of genes searched
CAAATG	191	73	76
CAACTG	74	47	76
CAAGTG	94	47	76
CAATTG	162	63	76
CACATG	155	63	76
CACCTG	35	25	76
CACGTG	56	24	76
CACTTG	94	47	76
CAGATG	85	45	76
CAGCTG	56	24	76
CAGGTG	35	25	76
CAGTTG	74	47	76
CATATG	156	54	76
CATCTG	85	45	76
CATGTG	155	63	76
CATTTG	191	73	76
