# Supplementary material for: A Nuclear Calcium-Sensing Pathway Is Critical for Gene Regulation and Salt Stress Tolerance in Arabidopsis
Source: PLoS Genet. 2013 Aug 29;9(8):e1003755. doi: 10.1371/journal.pgen.1003755 (PMC3757082; doi:10.1371/journal.pgen.1003755)
Supplement: Table S6 — Primers used in this study. (A) Primers used for ChIP-qPCR in this study. (B) Remaining primers used in this study. (PDF) [file pgen.1003755.s017.pdf]

Table S6. Primers Used in This Study.

Table S6A. Primers Used for ChIP-qPCR in This Study.

Name	Sequence (5' to 3')	Position (relative to translation start site)	*cis* element (for bHLH TF)	Purpose
SOS1 ChIP A 5'	CATTAGGATCGACGGTTGGTCGA	-984 to -882	CACTTG	ChIP-qPCR
SOS1 ChIP A 3'	TCATTTTCATGCTACCCAGAAGCACCT			
SOS1 ChIP B 5'	ATGTATTTTTGGTAGGCTAGTCGTCT	-643 to -497	CATTTG, CAATTG	ChIP-qPCR
SOS1 ChIP B 3'	TCGTAGTTGGTTTTTTGACATTTTGGGAC			
SOS1 ChIP C 5'	CATTGAAGAATTGGTCGGCTGAAAATTGTG	-235 to -31	CACGTG	ChIP-qPCR
SOS1 ChIP C 3'	CAGAGGAAGAAGAAGAATGCTCGTTCACTGA			
SOS1 ChIP D 5'	ACGACTGTAATCGACGCGACG	+4 to +128	not applicable	ChIP-qPCR (negative control)
SOS1 ChIP D 3'	AGAGACATTCCGACGAAGAGAACG			
At2g36690 ChIP A 5'	TGCGATAGAGTATGGAAGTACTGGCAATAG	-1356 to -1158	CAGTTG	ChIP-qPCR
At2g36690 ChIP A 3'	CGTCGCATATTATTGGGACGAGAATACCAG			
At2g36690 ChIP B 5'	CTTACCTTTTCGAACGAATCTAAAACGTGTCC	-923 to -730	CAAATG	ChIP-qPCR
At2g36690 ChIP B 3'	TGGGTTTAGAGAGTATTTTTTCTCCCGA			
At2g36690 ChIP C 5'	GAAGGAGACGTGAGCAAGAATATGATAGACG	+609 to +737	not applicable	ChIP-qPCR (negative control)
At2g36690 ChIP C 3'	CTGGTTGAAACTCGTGCCGTATCGTACTG			
Atg514130 ChIP A 5'	GCTTCCGGCACTGAGCAGAGCAGAAG	-1469 to -1356	CAACTG	ChIP-qPCR
Atg514130 ChIP A 3'	AGGATCCAGTTAATAAACCGGAGCGGAG			
Atg514130 ChIP B 5'	TCCTTTCCAACATGTTTCGAACCTAGC	-1196 to -985	CACTTG, CAGTTG	ChIP-qPCR
Atg514130 ChIP B 3'	TCCATATTACATGATCAACATTCTAGCTATCTG			
Atg514130 ChIP C 5'	GTATTCCGCTGAATTCATGGCGATCAGTC	- 525 to -214	CAGATG	ChIP-qPCR
Atg514130 ChIP C 3'	TCTAATCACATTCACAGGTGAATGCCAG			
Atg514130 ChIP D 5'	TCGATCACGATGGATATTCGTAGTGATG	+31 to +127	not applicable	ChIP-qPCR (negative control)
Atg514130 ChIP D 3'	CATTTGACTCGGCCACCATGGAAAAC			
At2g39040 ChIP A 5'	GTGGGCCTTTACCTTGTGGACTTAG	-833 to -588	CATATG (2 copies at two different sites)	ChIP-qPCR
At2g39040 ChIP A 3'	TCCATATGCAATGGGAATTTTGGGA			
At2g39040 ChIP B 5'	ACAGGGAACTTTGCTTGGCCACCCAGAC	-143 to +6	CAATTG, CAAGTG	ChIP-qPCR
At2g39040 ChIP B 3'	AGCCATGTGATCGACTTTAGATTTG			
At2g39040 ChIP C 5'	AGGAAGATGGGAAGGTAAGCTGAAGATG	+171 to +269	not applicable	ChIP-qPCR (negative control)
At2g39040 ChIP C 3'	GCTTCGACTTTCTTCCACACTATCTGC			

Table S6B. Remaining Primers Used in This Study.

Name	Sequence (5' to 3')	Purpose
RSA1 164 F	CACCTCCCCCGGGTGCAGTTGAGAAAAAATGTAAATA	pRSA1:GUS
RSA1 164 R	CGGGGTACCCTGGTTTCAATTTTGTAATTTTAG	
RSA1 104 F	CACCATGCATAGAGATATGTATTCTTCAAGAGG	p35S:YFP-RSA1
RSA1 104 R	CTATAGAGACAATCTAACAAGTTTATTGTA	
RSA1 99 F	CACCTCCCCCGGGTGCAGTTGAGAAAAAATGTAAATA	pRSA1:RSA1 in pMDC99
RSA1 99 R	CGAGCTCCTATAGAGACAATCTAACAAGTTTA	
RSA1 3507 attB1 F3	GGGGACAAGTTTGTACAAAAAAGCAGGCTTCCCTGAGGAGGATCCTGAAGAAGACCCTGA	pDEST17-RSA1
RSA1 CDS attB2 R	GGGGACCACTTTGTACAAGAAAGCTGGGTCCTATAGAGACAATCTAACAAGT	
RSA1genomic attB1 F	GGGGACAAGTTTGTACAAAAAAGCAGGCTTCTGCAGTTGAGAAAAAATGTAAATAAGTTG	pRSA1:RSA1-GFP in pMDC107
RSA1genomic attB2 R	GGGGACCACTTTGTACAAGAAAGCTGGGTCTAGAGACAATCTAACAAGTTTATTG	
RSA1 attB1 F	GGGGACAAGTTTGTACAAAAAAGCAGGCTTCATGCATAGAGATATGTATTCTTC	pDEST32-RSA1
RSA1 attB2 R	GGGGACCACTTTGTACAAGAAAGCTGGGTCCTATAGAGACAATCTAACAAGT	
RITF1 attB1 F	GGGGACAAGTTTGTACAAAAAAGCAGGCTTCATGGCGTCTCTGATCTCAG	pDEST22-RITF1
RITF1 attB2 R	GGGGACCACTTTGTACAAGAAAGCTGGGTCTCAAATCGGTGGAGGAGCTG	
RITF1 attB1 F	GGGGACAAGTTTGTACAAAAAAGCAGGCTTCATGGCGTCTCTGATCTCAG	p35S:RITF1-GFP
RITF1 GFP R	GGGGACCACTTTGTACAAGAAAGCTGGGTCAATCGGTGGAGGAGCTGAG	
RSA1 Xba I F	AGTCTCTAGAATGCATAGAGATATGTATTCTTCAAGAGG	pUC-SPYNE-RSA1
RSA1 Sal I R	ACTGGTCGACTAGAGACAATCTAACAAGTTTATTGTATAAAATTCG	
RITF1 BamH I F	ACTGGGATCCATGGCGTCTCTGATCTCAGATATTG	pUC-SPYCE-RITF1
RITF1 Sal I R	ACTGGTCGACAATCGGTGGAGGAGCTGAG	
At317100 AttB1 F	GGGGACAAGTTTGTACAAAAAAGCAGGCTTCATGGAGTCTATATCTCCGGTATCGAATC	pDEST22-At3g17100
At3g17100 AttB2 R	GGGGACCACTTTGTACAAGAAAGCTGGGTCCTAACCAAGCATGTGTGTTTGTCC	
At1g09250 AttB1 F	GGGGACAAGTTTGTACAAAAAAGCAGGCTTCATGGTGGAGTCTCTGTTCCCGAGCATC	pDEST22-At1g09250
At1g09250 AttB2 R	GGGGACCACTTTGTACAAGAAAGCTGGGTCTTAAGTTCCGGTCAACGTCGTCCGT	

At3g05800 AttB1 F	GGGGACAAGTTTGTACAAAAAAGCAGGCTTCATGTCTT CTGAACAAGGAAATGGATC	pDEST22-At3g05800
At3g05800 AttB2 R	GGGGACCACTTTGTACAAGAAAGCTGGGTCCTATAAA GCCGAGCCGAGATTAGTAGAC	
RSA1 3507 attB1 F3	GGGGACAAGTTTGTACAAAAAAGCAGGCTTCCCTGAGGAGGATCCTGAA GAAGACCCTGA	Three-step overlapping PCR amplifications of RSA1 fragment without EF-hand motif in pDEST17: step 1 using F3 and R1; step 2 using F3 and R2 (product from step 1 as a template); step 3 using F3 and R3 (product from step 2 as a template).
RSA1 w/o EF motif R1	GGGGACCACTTTGTACAAGAAAGCTGGGTCCA CGGTGAGATAGAAACTTTCCCAAGCTGTGAATGGTTACTCTCATAAAAAA TCTGAAAG	
RSA1 w/o EF motif R2	GGGGACCACTTTGTACAAGAAAGCTGGGTCCAGTGT TGCTCTCTAACAAAGCGCTCTGCACAAGTTCCTTGACTTCACGGTGAGATA GAA	
RSA1 w/o EF motif R3	GGGGACCACTTTGTACAAGAAAGCTGGGTCCTA AGACAATCTAACAAGTTTATTGTATAAAATTCGATCATCTCTTCCAGTGTT GCTCTC	
RSA1 attB1 F	GGGGACAAGTTTGTACAAAAAAGCAGGCTTCATGC ATAGAGATATGTATTCTTC	p35S:YFP-RSA1(-EF). Three-step overlapping PCR amplifications: step 1 using F and R1; step 2 using F and R2 (product from step 1 as a template); step 3 using F and R3 (product from step 2 as a template).
RSA1 w/o EF motif R1	GGGGACCACTTTGTACAAGAAAGCTGGGTCCA CGGTGAGATAGAAACTTTCCCAAGCTGTGAATGGTTACTCTCATAAAAAA TCTGAAAG	
RSA1 w/o EF motif R2	GGGGACCACTTTGTACAAGAAAGCTGGGTCCAGTGT TGCTCTCTAACAAAGCGCTCTGCACAAGTTCCTTGACTTCACGGTGAGATA GAA	
RSA1 w/o EF motif R3	GGGGACCACTTTGTACAAGAAAGCTGGGTCCTA AGACAATCTAACAAGTTTATTGTATAAAATTCGATCATCTCTTCCAGTGTT GCTCTC	
RSA1 attB1 F	GGGGACAAGTTTGTACAAAAAAGCAGGCTTCATGC ATAGAGATATGTATTCTTC	RSA1-nLUC for Split-LUC assay
RSA1genomic attB2 R	GGGGACCACTTTGTACAAGAAAGCTGGGTCTAGAG ACAATCTAACAAGTTTATTG	
RITF1 attB1 F	GGGGACAAGTTTGTACAAAAAAGCAGGCTTCAT GGCGTCTCTGATCTCAG	RITF1-cLUC for Split-Luc assay
RITF1 GFP R	GGGGACCACTTTGTACAAGAAAGCTGGGTC AATCGGTGGAGGAGCTGAG	
SOS1 pro kpnI F	AGTCGGTACCCGTAAGAAACTTGTGAATGGCAGAGGCAAGTG	SOS1:LUC for dual luciferase reporter assay
SOS1 pro speI R	AGTCACTAGTGAAGCAACAACACAGAGGAAGAAGAAGAATGCTCG	
RITF1 CDS speI F	AGTCACTAGTATGGCGTCTCTGATCTCAG	RITF1 for dual luciferase reporter assay
RITF1 CDS kpnI F	AGTCGGTACCTCAAATCGGTGGAGGAGCTG	
RSA1CDS xbaI F	AGTCTCTAGAATGCATAGAGATATGTATTCTTCAAGAGGCACTGG	RSA1 for dual luciferase reporter assay
RSA1CDS kpnI F	AGTCGGTACCCTATAGAGACAATCTAACAAGTTTATTG	
RSA1CDS xbaI F	AGTCTCTAGAATGCATAGAGATATGTATTCTTCAAGAGGCACTGG	RSA1(-EF) for dual luciferase reporter assay
RSA1CDS kpnI F	AGTCGGTACCCTATAGAGACAATCTAACAAGTTTATTG	
SOS1 attB1 F	GGGGACAAGTTTGTACAAAAAAGCAGGCTTCATGACGACTGTAATCGACG CGACGATGG	p35S:HA-SOS1

SOS1 attB2 R	GGGGACCACTTTGTACAAGAAAGCTGGGTCTCATAGATCGTTCCTGAAAACGATTTTAC	p35S:HA-SOS1
At3G06590 qPCR F	CTCTGCTTCATCCGCCGCATCGTCTC	qRT-PCR
At3G06590 qPCR R	GAGCTCGATCGGCGGCTTCACGGACG	
At2g39040 qPCR F	AGCTGCCCTGGAGCCGAGGATATC	qRT-PCR
At2g39040 qPCR R	TGATTTCAAACCCGGAAAGCGAG	
RSA1 qPCR F	GAAGTTCAGTGAGTGGTGGCGCAGAAGGAG	qRT-PCR
RSA1 qPCR R	GGTGAATCAGGTAAAGTAGGGACATATC	
At2G36690 qPCR F	CAAGTATATATGGCCTGAACCTGACCGA	qRT-PCR
At2G36690 qPCR R	CATGATTCACAACCTGAAAGAAGCCGTAG	
rsa1-3 qPCR F	CAGATTTTTTGACCGTAACCAGGCA	qRT-PCR
rsa1-3 qPCR R	CTAACAAAGCGCTCTGCACAAGT	
rsa1-2 qPCR F	AAGGACACAAAGGAGCGGACT	qRT-PCR
rsa1-2 qPCR R	ATGTCAACCAATCGCGAAGA	
SOS1 qPCR F	GACGCCGTTCTCTTCGTCGGAATGTCTC	qRT-PCR
SOS1 qPCR R	GAGAGATCCAAGAGCAATTCCGATAACG	
At5g14130 qPCR F	TGGTGGCCGAGTCAAATGCTC	qRT-PCR
At5g14130 qPCR R	TGTCATCTGCGTCTTTCTCTGCGTC	
At1g10480 qPCR F	AGTCGTACGCCGGAAATCACG	qRT-PCR
At1g10480 qPCR R	TGCATGGAGGAGAATGCACAATACG	
At5g67400 qPCR F	TGACACAACCACTGCTCAACTC	qRT-PCR
At5g67400 qPCR R	ACGTCAAATCCATCTCCGGCCAAAG	
At3g56980 qPCR F	TCCAAACTTTGGGTGGCCATCAAC	qRT-PCR
At3g56980 qPCR R	AGCATTGTGATTAAGCTTCTTGACGACC	
At3g17100 qPCR F	GAGCTCTAATTCCGATCGCTCTCGTC	qRT-PCR
At3g17100 qPCR R	CTTCACGTACGGCTCTACCTCCACGTGG	
At1g09250 qPCR F	GAGATAGAGGCACGACGTGTCAACGAAG	qRT-PCR
At1g09250 qPCR R	CACCGAGTCGTACCACGAGCGGACG	
At3g05800 qPCR F	GTTACCGGAGCTTTTGGACGAGACAG	qRT-PCR
At3g05800 qPCR R	CATAGTATTTTCGTCCAACTCGTGA	
TUB 8 F	ATAACCGTTTCAAATTCTCTCTCTC	qRT-PCR
TUB 8 R	TGCAAATCGTTCTCTCCTTG	
CS16026-LP	CTGAAGCCGAAACTGATAATAGA	Genotyping
CS16026-RP	GAAACTCGCAAGATTACTGGTTA	
CS811403 LP	GCATTACAAATGCCACTTTCG	Genotyping
CS811403 RP	TTAACCTCATCAAATCGGTGG	
SALK_007142 LP	AGAAGCGATGCTTGATCAATGCGATC	Genotyping
SALK_007142 RP	AGTCTCTTCCTCTTCTCTCAACACG	
GK233G09 LP	GGTTAGTCGATTCGGAACGAGTC	Genotyping
GK233G09 RP	ACTGGTGGAACCTAGATTCTTG	
GK428G06 LP	CCACATGAATTCTACCTCAACC	Genotyping
GK428G06 RP	ACTCGCTAAAATCGCTCTGCTC	
SALK_022587 LP	CAGAGAAAACGCATAACCCAG	Genotyping
SALK_022587 RP	TTTCTCCAACGATATTGACGG	
